# Unraveling the complexity of human behavior and urbanization on community vulnerability to floods

**DOI:** 10.1038/s41598-021-99587-0

**Published:** 2021-10-11

**Authors:** Mona Hemmati, Hussam N. Mahmoud, Bruce R. Ellingwood, Andrew T. Crooks

**Affiliations:** 1 Lamont-Doherty Earth Observatory, Columbia University, Palisades, NY USA; 2 Department of Civil and Environmental Engineering, Colorado State University, Fort Collins, CO USA; 3 Department of Geography, University at Buffalo, Buffalo, NY USA

**Keywords:** Natural hazards, Civil engineering

## Abstract

Floods are among the costliest natural hazards and their consequences are expected to increase further in the future due to urbanization in flood-prone areas. It is essential that policymakers understand the factors governing the dynamics of urbanization to adopt proper disaster risk reduction techniques. Peoples’ relocation preferences and their perception of flood risk (collectively called human behavior) are among the most important factors that influence urbanization in flood-prone areas. Current studies focusing on flood risk assessment do not consider the effect of human behavior on urbanization and how it may change the nature of the risk. Moreover, flood mitigation policies are implemented without considering the role of human behavior and how the community will cope with measures such as buyout, land acquisition, and relocation that are often adopted to minimize development in flood-prone regions. Therefore, such policies may either be resisted by the community or result in severe socioeconomic consequences. In this study, we present a new Agent-Based Model (ABM) to investigate the complex interaction between human behavior and urbanization and its role in creating future communities vulnerable to flood events. We identify critical factors in the decisions of households to locate or relocate and adopt policies compatible with human behavior. The results show that when people are informed about the flood risk and proper incentives are provided, the demand for housing within 500-year floodplain may be reduced as much as 15% by 2040 for the case study considered. On the contrary, if people are not informed of the risk, 29% of the housing choices will reside in floodplains. The analyses also demonstrate that neighborhood quality—influenced by accessibility to highways, education facilities, the city center, water bodies, and green spaces, respectively—is the most influential factor in peoples’ decisions on where to locate. These results provide new insights that may be used to assist city planners and stakeholders in examining tradeoffs between costs and benefits of future land development in achieving sustainable and resilient cities.

## Introduction

Floods are among the costliest natural hazards and threaten the lives and livelihoods of millions of people worldwide^1–5^. The annual cumulative losses due to various types of flooding are higher than those from large-scale disasters such as earthquakes^6^. The consequences of flooding are intensified by climate change and socioeconomic development. Urbanization, as a direct result of socioeconomic development, exposes more people and their livelihoods to risk^7–12^. The United Nations has projected that 68% of the world’s population will live in urban areas in 2050, compared to 50% in 2020^13^. Urbanization occurs mainly in low-lying, flood-prone areas due to accessibility to recreational facilities and agricultural development^14^. Therefore, the interaction of more intense and frequent flooding events due to climate change with the increased exposure brought by urbanization may lead to catastrophic social and economic consequences in the future if not addressed^12,15,16^. As a result, the United Nations has emphasized the importance of sustainable development by building resilient cities and communities subjected to natural hazards including floods, as its 11th Sustainable Development Goals (SDGs)^17^.

People and their perception of flood risk—collectively, human behavior—is one of the most significant factors influencing urbanization. Therefore, achieving sustainable development requires a comprehensive understanding of human behavior and its impact on flood risk^18^. Households, real estate, developers, government, and their interactions can create favorable or unfavorable socioeconomic conditions and consequently encourage or discourage demand in specific areas that shapes the city's expansion. On the other hand, an effective Disaster Risk Reduction (DRR) plan requires a reliable risk assessment^18^. Rising social and economic losses from flooding events have demonstrated that current governmental investments in adaptation measures are insufficient owing to the dynamic nature of risk, which is due, in part, to the failure to consider human behavior in flood risk assessments^18–20^. While some studies have focused on conceptualizing human-flood interaction^18,21^, they have not suggested quantitative approaches to employ engineering techniques to measure the role of human behavior in future vulnerabilities to flood and what management strategies should be used to mitigate flood impacts. To steer urbanization toward sustainable development and resilient cities and communities in flood events, human behavior impacts on urbanization and flood risk must be thoroughly understood and quantified.

Policymakers can invest in two types of strategies to cope with rising flood risk: structural or nonstructural strategies^11,12^. Structural measures typically involve engineered systems, such as dams, levees, and floodwalls aimed at *controlling the hazard*. If not carefully evaluated, these interventions may cause detrimental impacts on human lives and livelihoods, as well as on the biophysical environment. For instance, studies have demonstrated that the construction of polders has increased the extent of pluvial flooded areas in Bangladesh^22–24^. These strategies may create an illusion of safety which promotes further growth in or near flood-protected areas^25^. Moreover, with the increasing trends in frequency and intensity of flood events, protective structures that are designed based on contemporary flood threats may experience future extreme events that cause overtopping or failure of the structure and threaten the lives of people who live in the protected area^26^. Nonstructural flood mitigation strategies, on the other hand, rely on public policy planning, such as zoning, acquisition and land-use regulation, and socioeconomic incentives that focus on *controlling the exposure*. These policies are most effective in developing communities, where such choices are more practical than in established communities with fixed infrastructure and can shape urban expansion over an area effectively. However, they are not as successful as expected in reversing the tendency of people to choose to live in flood-prone areas^27^. Consequently, there is an essential need to consider human behavior and their locational living preferences in implementing these nonstructural measures in rapidly developing urban areas.

In this article, we demonstrate how changes in human behavior affect urbanization and vulnerabilities to flooding in expanding communities. We first show how human behavior impacts urbanization by developing a novel ABM, in which households, real estate, and developers are modeled as agents, the interactions of which lead to changes in the urban expansion of a community over time. Next, we evaluate how the risk perception of households affects their decisions in where to locate. Finally, using the ABM approach, we investigate the driving factors and incentives in the human decision on locational choices and how these incentives can be imposed on the communities by local authorities and policymakers as nonstructural flood mitigation measures to shape urbanization achieving sustainable development and resilient cities and communities in flooding events.

## Coupling human behavior, urbanization, and flood risk

**Figure 1:**
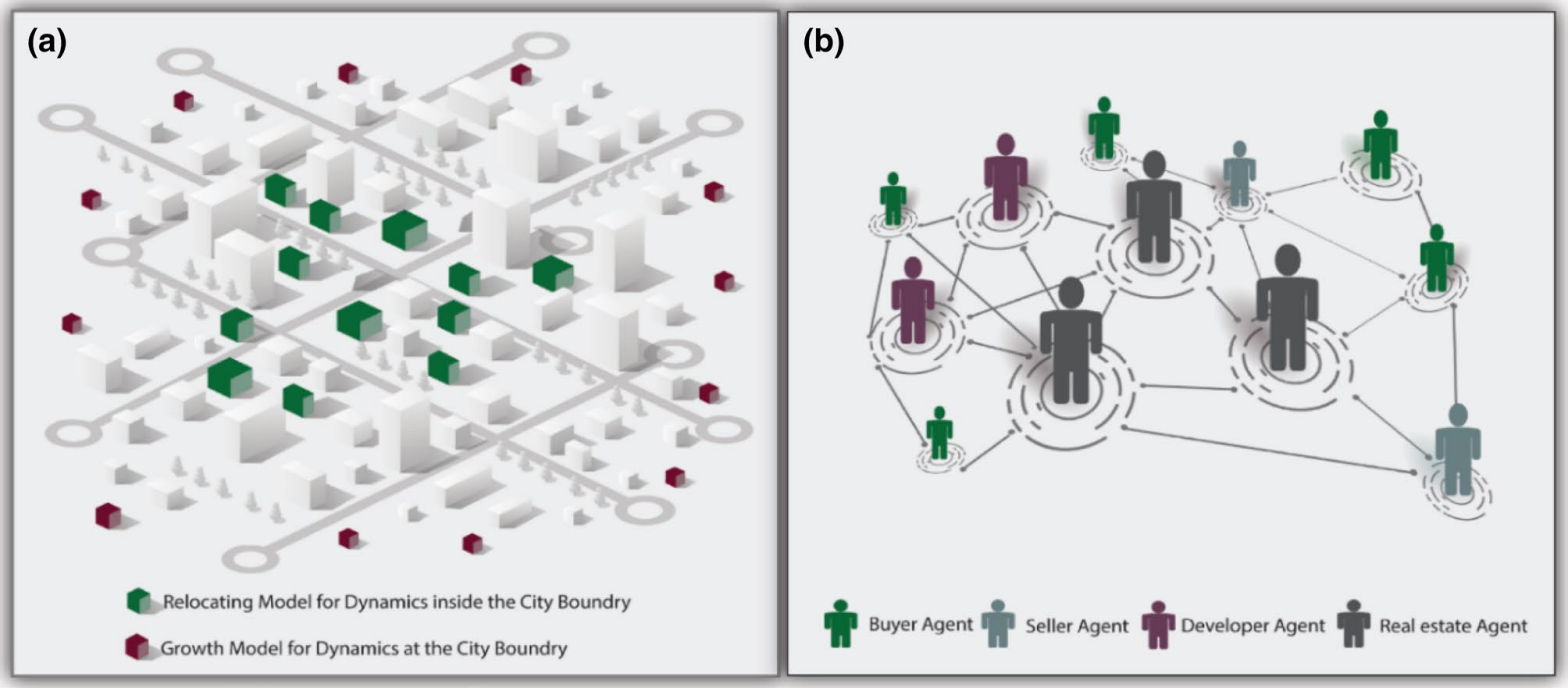
Conceptual model of the proposed behavioral urban growth model.

Urbanization occurs as a result of two processes: urban dynamics *inside* the city boundary and urban dynamics *at* the city boundary, each of which is influenced by individuals’ behavior. Some individuals who seek housing prefer to live within the city boundary while others select newly developed housing in the suburbs. Therefore, modeling these two processes is essential in understanding the role of human behavior in urbanization. Herein, we develop a behavioral urban growth model that employs ABM as a class of computational models for simulating the interactions between autonomous entities in the form of agents to investigate their consequences on complex systems and networks^28^. Our behavioral urban growth model consists of two sub-models: the *Relocating Model* which simulates the dynamics within the city boundary and the *Growth Model* which mimics the dynamics at the city boundary, as illustrated in Fig. 1. These two sub-models are connected through the supply and demand of the real estate market. Further information is given in “Material and methods” section of the paper and in Supplementary Information.

As the interaction of sellers and buyers in the real estate market shapes the dynamic inside the city boundary, the Relocating Model (see Figure S1) consists of three agents: real estate agent, seller agent, and buyer agent. The real estate agent provides an estimation of housing price using a hedonic price model^29^ and assists the negotiation process (see Figure S2) between sellers and buyers. This agent can capture the effect of flood risk on the real estate market by modifying the hedonic price model. The seller agent is the other effective entity in the Relocating Model that participates in this process by providing housing options within the current city limit and adding them to the real estate market as available choices. The seller decides to relocate or leave the study region with the aim to maximize its profit. In this study, we have not evaluated the effect of flood risk on the decision of the seller to relocate. Therefore, the seller agent adds its housing unit to the market regardless of its intention. The buyer agent is the third component of the Relocating Model. Accordingly, buyers represent the households who seek housing that can maximize its expediency. Whether or not buyers consider the flood risk on their decision, they decide where to locate according to two behaviors: Risk-Negligence and Expected Utility^30^. Individuals with Risk-Negligence behavior do not consider flood risk in their decision as they may or may not be aware of such risk. On the other hand, individuals who take the Expected Utility attitude aim at maximizing their utility while considering future flood risk. The information regarding modeling the behavior of real estate agent, seller agent, and buyer agent is provided in “Material and methods” section.

The Growth Model, on the other hand, simulates the process of converting undeveloped lands at the city boundary to developed lands, which causes the expansion of the community over time. This process occurs as a result of interaction between developers and buyers in the real estate market. Hence, this sub-model, implemented using Cellular Automata (CA) (see Figure S3), also consists of three main agents: real estate agent, developer agent, and buyer agent, each of which has been described in detail in “Material and methods” section and Supplementary Information. The developer agent can also show two types of behaviors: developing under risk (Risk-informed Behavior) and developing without risk (Normal Behavior), based on the flood risk and the expected return from buying undeveloped lands. These behaviors are also comprehensively described in “Material and methods” section.

Using the aforementioned urban growth model, we propose a framework that accounts for the role of urbanization, influenced by human behavior, on future flood risk. The overall framework consists of fully integrated models: (1) behavioral urban growth, (2) flood hazard, and (3) policy implementation. In this study, we defined two potential nonstructural flood mitigation measures in terms of socioeconomic incentives. These measures include Policy I: building new educational facilitates and shopping centers in the northern part of the city, and Policy II: creating green spaces and water bodies in the southern part of the city. The details of each model and the aforementioned policies can be found in “Material and methods” section. Using this framework, effective nonstructural flood risk mitigation policies, in terms of socioeconomic incentives, can be devised that take human behavior into account. Such policies are more likely to be accepted by the community and will be more effective in shaping urbanization toward safer and less vulnerable areas to floods. To the best of our knowledge, this is the first framework that incorporates the urbanization dynamics both *inside* and *at* a city boundary. This point will assist us in evaluating the role of human behavior in shaping urban growth explicitly and at a very high resolution.

## Model application

**Figure 2:**
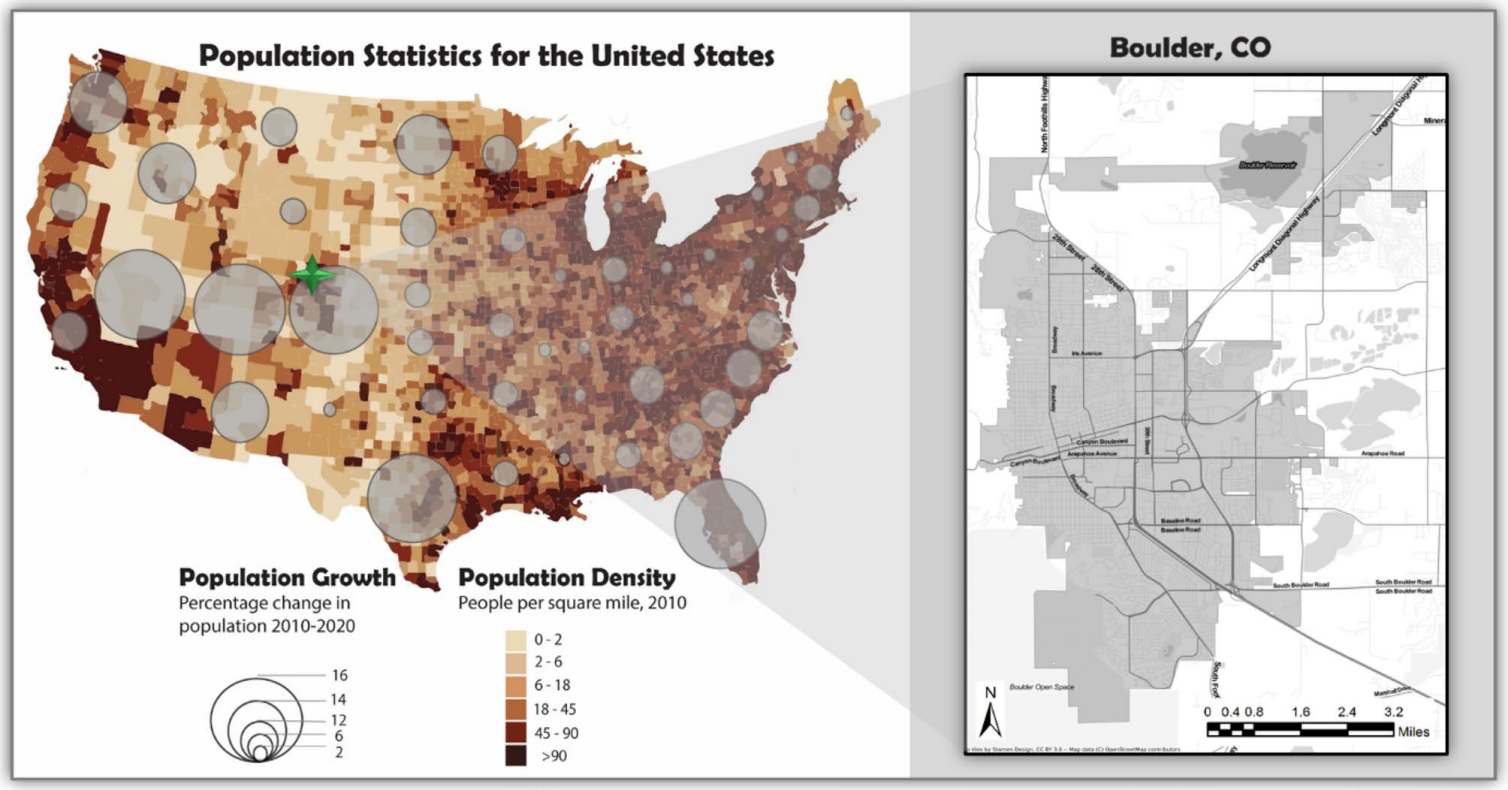
Population statistics in the United States and Boulder, the testbed of this study—figures created using Python 3.9.5 (https://www.python.org/doc/).

The City of Boulder, shown in Fig. 2, was selected as the testbed of this study. Boulder is located in Colorado which is among the states that have experienced a high rate of population growth from 2010 to 2020. Boulder is an upper middle-class community with approximately 100,000 inhabitants^31^. Home to a world-class research university, a mix of key industry clusters, major government research facilities, visionary entrepreneurs, and a highly educated population, Boulder has experienced a significant urban expansion in the past half-century due to population and economic growth. Boulder is susceptible to flooding due to its geographical location on the Eastern Front Range of the Rocky Mountains, which contains many streams and creeks. The City’s assessment of exposure to flood risk reveals that there are about 10,000 people and 3,600 structures with an assessed valuation of $1 billion within Boulder’s 100-year floodplain^31^. The City has imposed restrictive regulations in new development and redevelopment activities. However, the city is still expected to continue to grow as the population estimate for the year 2040 is about 123,000 inhabitants. If this growth occurs within floodplains, it poses further potential risks by adding more exposure to floods and changing the floodplains. Current regulations do not restrict the redevelopment of properties within floodplains but do require suitable structural and/or nonstructural flood protection. However, these properties would still be subject to flood damage from larger flood events that appear plausible under the effects of climate change. In this study, we estimated urban growth using the CA model, according to the steps outlined in Figure S4, in Supplementary Information.

## Effect of flood risk on behavior of driving agents in urbanization

Using the proposed behavioral development framework summarized above, we seek to evaluate the changes in urbanization caused by changes in the behavior of the key actors (e.g., developers, buyers, and sellers), as influenced by flood risk. We first assess the effect of floods on the real estate agent to investigate how flood risk can change the housing prices in the market. Next, we examine how vulnerabilities to floods influence buyers’ decisions on where to locate. We perform statistical analysis to identify the factors that most influence buyers’ decisions on their locational choices. Finally, we employ this information to identify policies that (a) promote urbanization toward less vulnerable areas to floods in the community using socioeconomic incentives and (b) gradually encourage people to relocate to safer regions without forcing them to do so, unlike policies such as acquisition and buyout.

### Effect of flood risk on the real estate agent

**Figure 3:**
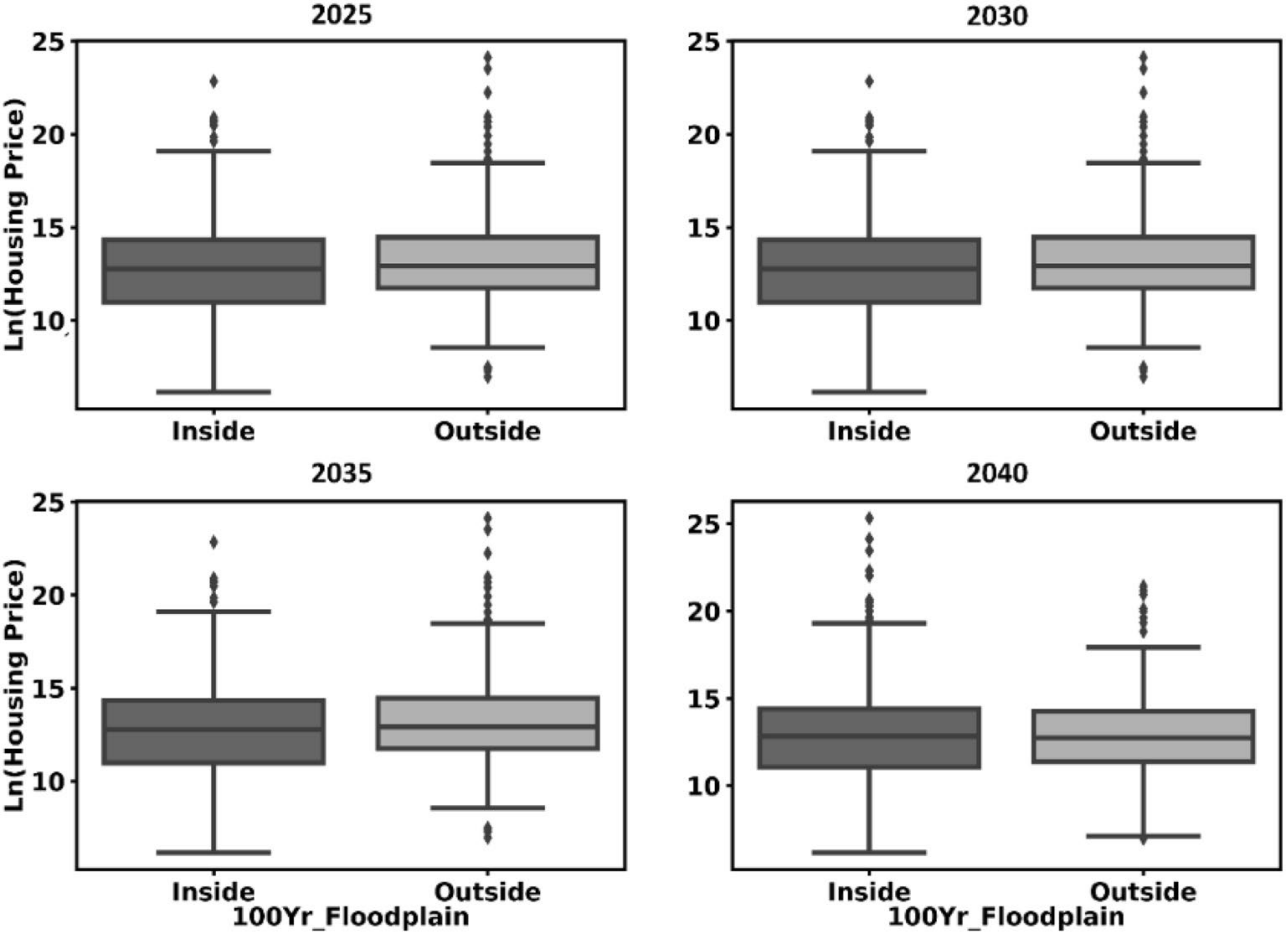
Projection of sale transactions (in natural log) from 2020 to 2040 for housing inside and outside the 100-year floodplain.

As a first step, we performed two sets of analysis: (1) examination of historical sale transactions from 2010 to 2020 to train the ABM model, (2) prediction of the sale prices for 2020–2040, as illustrated in Fig. 3. These analyses were calculated by the real estate agent using the hedonic price model introduced in “Material and methods” section. The historical data revealed that the housing prices inside both 100-year and 500-year floodplains are lower than the housing prices outside the floodplains (note: the difference does not appear to be large in Fig. 3 because the prices are plotted as natural log). Since the real estate agent behavior was trained based on the historical data, future projections inside 100-year floodplain are expected to reveal the same trend, as shown in Fig. 3. The results of the historical analysis and the projection of sale transactions from 2020 to 2040 for housing inside the 500-year floodplain are presented in Supplementary Infor﻿mation (see Figures S5 and S6). This fact raises a question: whether these lower prices will ultimately promote settlement in the flood-prone areas compared to less vulnerable regions, which will result in rising flood risk. This question will be answered in the upcoming sections.

### Effect of flood risk on the buyer agent

**Figure 4:**
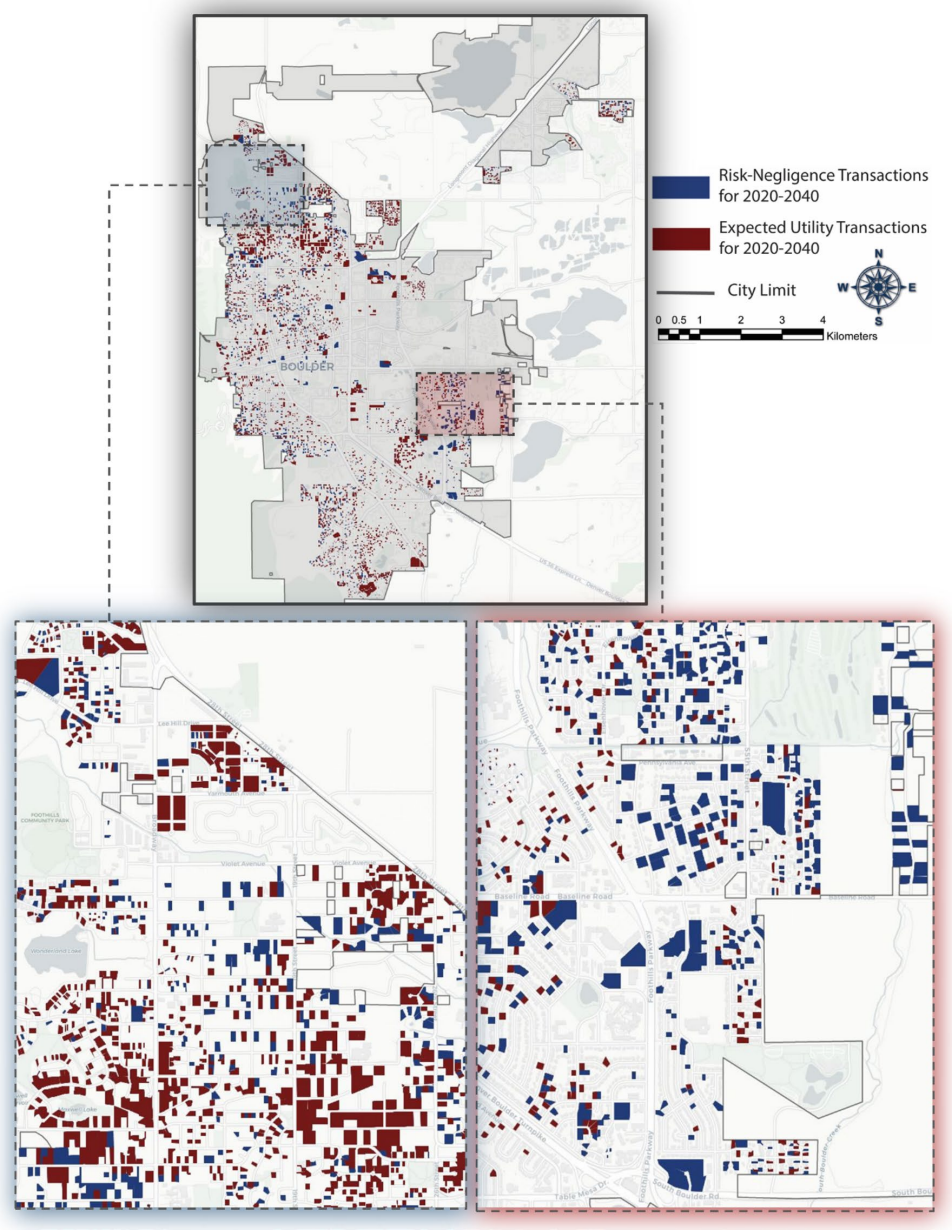
Future projections of buyers’ choices from 2020 to 2040—figures created using Python 3.9.5 (https://www.python.org/doc/).

We next performed analyses to assess how individuals’ decisions are influenced by flood risk. With this aim, we analyzed the buyers’ choices on a historical basis for the period of 2010–2017 under the two behaviors: Risk-Negligence and Expected Utility. We calibrated the model for this period by comparing the actual sale transactions to the results from the model for this historical period. To validate the model, the buyers’ decisions for the year 2020 were estimated and compared to the actual sale transactions in 2020. The results of historical analysis of the buyer choices under these two behaviors are presented in Supplementary Information (see Figure S7). Finally, we calculated the projections of sale transactions for 2020 to 2040, illustrated in Fig. 4, to investigate how these interactions between buyer, seller, and real estate agents would affect the vulnerability to future floods. As shown in Fig. 4, Eastern Boulder is more vulnerable to floods and this area is selected the most by Risk-Negligence Buyers due to lower housing prices inside the floodplains. However, the northern part of Boulder is less susceptible to floods and this area is selected by most Expected Utility buyers even though housing prices tend to be higher.

Table 1 summarizes the percentages of sales within 100-year and 500-year floodplains under these two behavior scenarios for the historical records and future projections. These results show that when the individual decision is simply based on price, implying Risk-Negligence behavior, it will result in more choices in floodplains and an increase in future flood risk because the housing prices in the floodplains are less than housing prices outside the floodplains. This fact will increase the popularity of such housing within the floodplain; policies such as buyout and acquisition would be more challenging for policymakers and local authorities to implement. As shown in Table 1, the projections of housing choice by buyers reveal that nearly 17% and 29% of the sale transactions under Risk-Negligence behavior are inside the 100-year and 500-year floodplains, respectively, while these numbers decrease to 2% and 15% if the choices are made based on the Expected Utility behavior. This means that if the individuals seeking housing are aware of the risk and make a fully informed decision having full knowledge about their choices and their consequences, future flood risk can be mitigated since exposure will be reduced. This information can be beneficial for policymakers to plan for enhancing the resilience of communities in floods, as will be explained in the next section.

**Table 1:** Percentage of each behavior observed within floodplains (Risk-Negligence (RN) and Expected Utility (EU)).

Floodplain/Behavior	Historical analysis (2010–2017)		Future projections (2020–2040)	
	RN (%)	EU (%)	RN (%)	EU (%)
100-year floodplain	15	< 1	17	< 2
500-year floodplain	22	8	29	15

### Policy implementation

As the previous analysis has indicated, individuals make more informed decisions in their housing choices regarding future risks when they consider flooding consequences on their locational choices. This will reduce the attraction of housing in those regions susceptible to floods. Therefore, the local authority can perform buyout and acquisition with less social and economic consequences. Thus, as a first policy toward enhancing the resilience of communities to floods, buyers seeking housing should be informed about potential future flood events and the social and economic consequences that they may incur in the future. This information can be provided through the real estate market or other educational programs. There have been some initiatives that prove the effectiveness of such programs. For example, First Street Foundation, a nonprofit organization that provides flood risk at U.S. national level, has collaborated with Realtor.com, a website that provides housing information to buyers. This work has resulted in revealing housing information along with their susceptibility to flood risk to buyers^32^. Such initiatives could gradually increase the knowledge of people about the flood risk and its consequences in a building’s lifetime.

On the other hand, when individuals adopt a Risk-Negligence attitude toward flood risk, they decide to purchase a home solely based on the housing price, which simply represents a bundle of building and environmental characteristics: acreage, building age, square footage, number of bedrooms, and neighborhood quality. In this study, neighborhood quality is defined as the accessibility of the area to highways, education facilities, city center, water bodies and lakes, parks, and green spaces. One may ask: which of these features have a higher impact on the decision of individuals? The answer can help planners to impose policies that are compatible with people’s behavior resulting in enhancing resilience in a community and using a bottom-up policymaking process to protect future generations in flooding events. The subsequent section will help to answer this question.

### Driving factors in buyers’ decisions

To adopt policies that are compatible with human behavior, we should have information about the factors that most influence buyers’ decisions on where to locate. Assuming that the buyers are not fully informed about the vulnerability of a property to flood risk, they decide their housing location solely based on housing price using a Risk-Negligence behavior, explained in “Material and methods” section. The housing price is a bundle of bedroom numbers, square footage, acreage, building age, and neighborhood quality. Here, we investigate the importance of each of these characteristics on housing price and consequently the buyers’ decision on their locational choices. Such information can help to adopt suitable socioeconomic incentives in less susceptible regions to shape the urbanization on creating sustainable communities and cities in floods. Accordingly, we performed three statistical tests to find the relative importance of the aforementioned housing characteristics on the housing price.

The first statistical test is an analysis of variance to investigate the different weights that the critical features—bedroom number, square footage, acreage, building age, and neighborhood quality—have over the housing prices. Using Eq. (3), explained in “Material and methods” section and trained for the buyer agent, the independent variables in this equation are excluded from the housing price model one by one and the change in the coefficient of determination (R^2^), which is a statistical measure indicating how much variation of a dependent variable is explained by the independent variable, is determined. The results, presented in Table 2, indicate that the neighborhood quality term has the highest impact on R^2^. In other words, the neighborhood quality term is more responsible for the housing price variation in comparison to the other factors considered above.

**Table 2:** Analysis of variance for the coefficient of determination of Risk-Negligence behavior of buyers.

Function	R-squared
Include all terms	0.994
Exclude SQFT	0.993
Exclude no. BedRm	0.994
Exclude Acreage	0.991
Exclude Age	0.991
Exclude Neighborhood Quality	0.906

**Figure 5:**
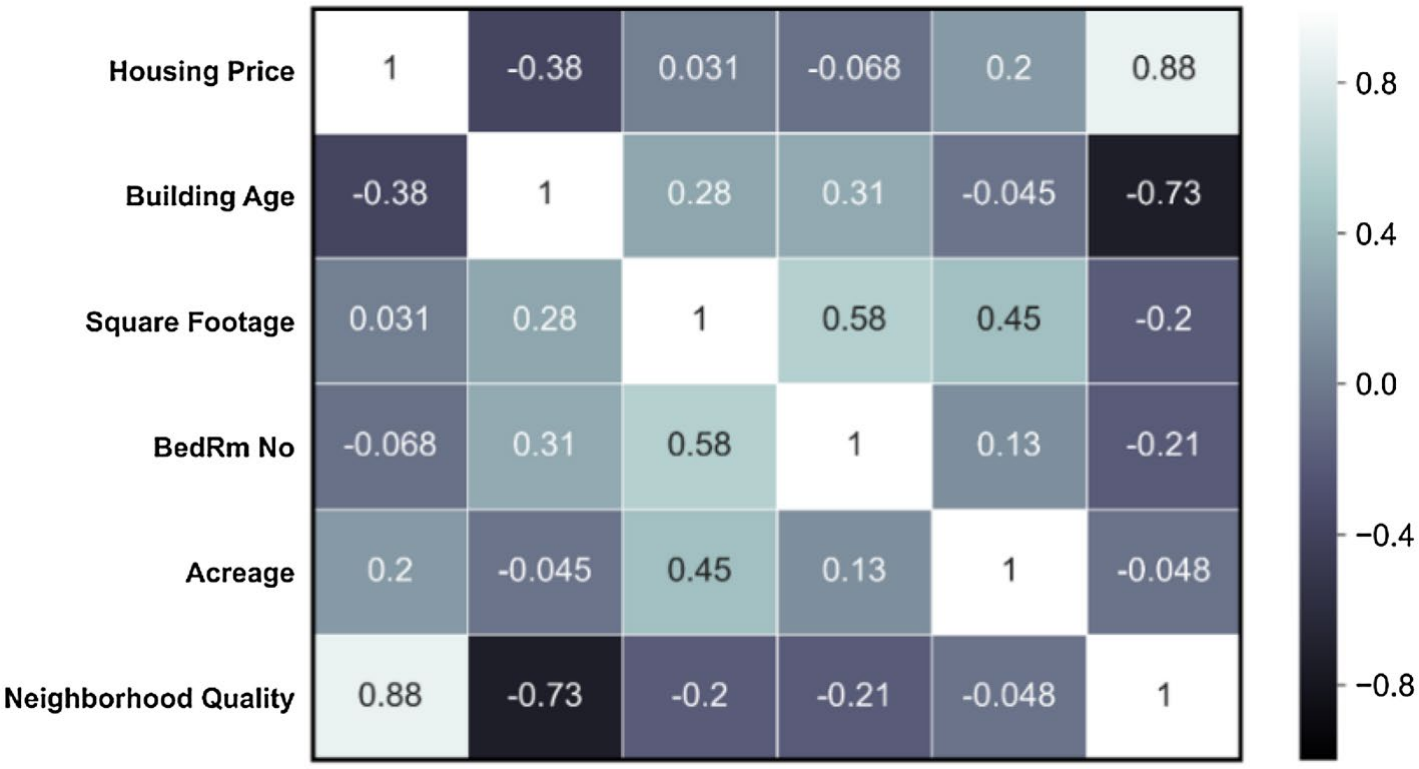
2D heat maps for housing price and variables in Risk-Negligence behavior of buyers.

The second statistical test involves a 2D heatmap, shown in Fig. 5 that indicates the correlation between the features influencing the Risk-Negligence behavior of the buyer agent and housing prices. As Fig. 5 illustrates, there is a strong positive correlation between the housing price and neighborhood quality, as found previously. The next most influential variable is building age, which has a negative correlation with the housing price. It is interesting to note that square footage and acreage are only weakly correlated to housing prices.

The third and final statistical test is a Principal Component Analysis (PCA), a dimensional reduction technique used to reveal strong patterns in a dataset^33^. In simple words, PCA is a method of extracting the most influential parameters in a dataset. First, the dataset needs to be standardized for overcoming scaling impacts and other features that may inaccurately affect the analysis. Second, the number of principal components is calculated by drawing a horizontal line at an eigenvalue equal to one; the number of components with an eigenvalue larger than one equals the number of main principal components. The PCA reveals that housing prices consist of two main principal components (PC), which are responsible for about 40% and 30%, respectively, of the variation in housing prices. The results of this analysis are provided in Supplementary Information (see Figure S8).

**Figure 6:**
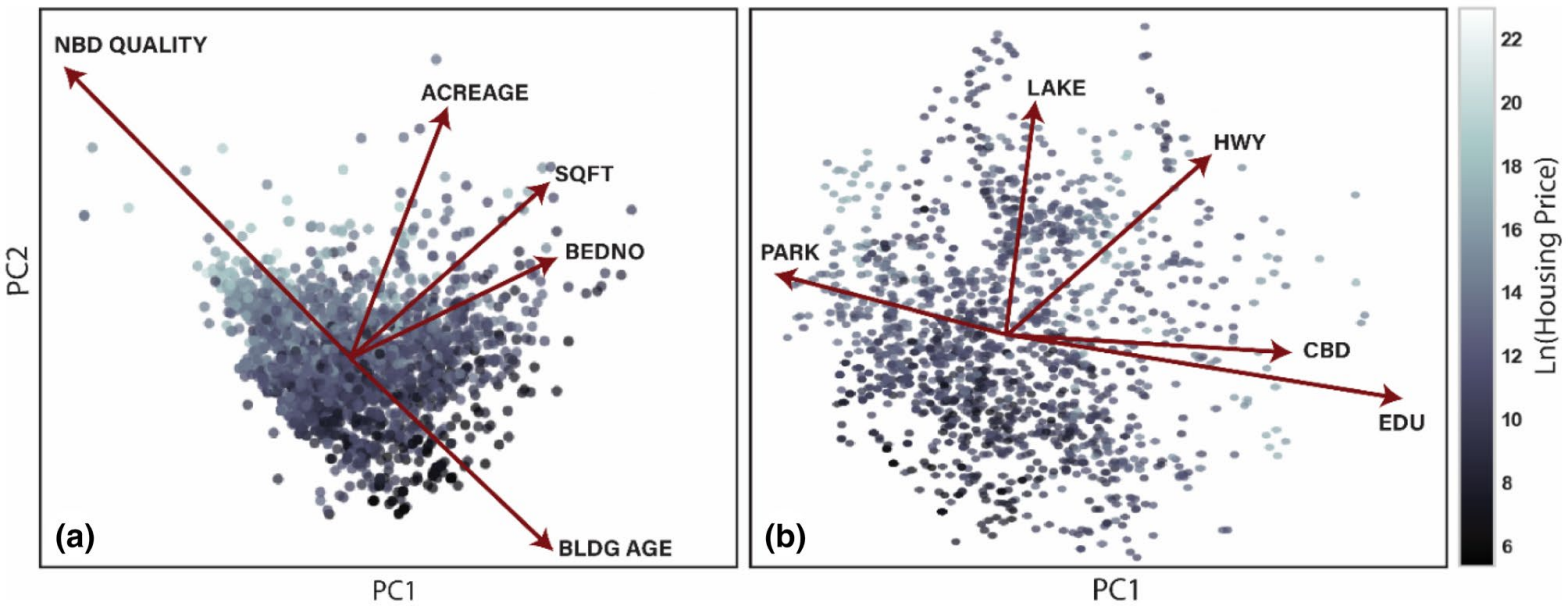
A biplots graph using the PCA analysis. The dots represent the housing prices and different colors are a range of prices on a logarithmic scale.

The PCA analysis was used to construct the biplot graph illustrated in Fig. 6, in which the size of each vector represents the importance of that variable on the housing prices, while the angles between the vectors describe the effect of each variable on the others, smaller angles indicate a stronger dependence. Moreover, the angle of each vector to the X- and Y-axes represents the importance of that feature on the main principal components. Figure 6a shows that neighborhood quality—defined as accessibility to highways, education facilities, city center, water bodies, and green spaces—is the characteristic among housing features that is most important. To identify the dominant contributor to neighborhood quality among these terms, we construct the second biplot graph, illustrated in Fig. 6b, using PCA. Figure 6b shows that, in rank order, accessibility to education centers, highways, city center, water bodies, and green spaces are the most critical features impacting neighborhood quality.

### Effect of flood risk on the developer agent

**Figure 7:**
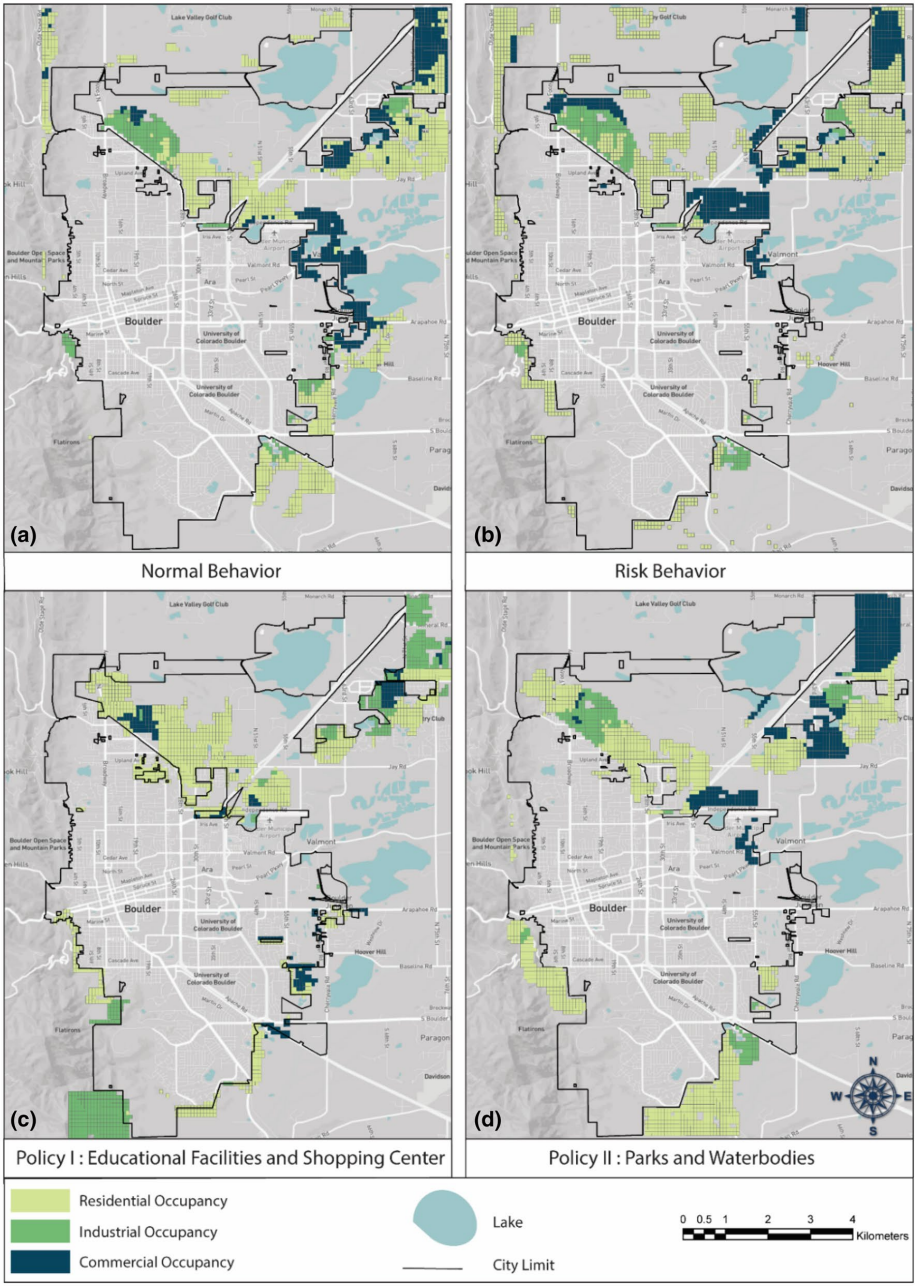
Growth projections for the City of Boulder, considering developer’s: (**a**) Normal Behavior, (**b**) Risk-informed Behavior, (**c**) Policy I, and (**d**) Policy II—figures created using Python 3.9.5 (https://www.python.org/doc/).

Our next step is to assess how flood risk affects the behavior of the developer agent and how the policies extracted from the previous step can be applied in the future urban expansion of the community by the developer agent *at* the city boundaries to achieve sustainable development. We evaluate the projected growth of Boulder for the year 2040 under the four different development scenarios identified in Table 3 and Fig. 7a–d. These scenarios are explained in detail in “Material and methods” section. These figures show that if the developer agent makes a risk-informed decision for buying undeveloped lands and converting them to developed lands, s/he selects northern Boulder instead of eastern Boulder for future development due to a higher expected return since the lands adjacent to eastern Boulder are more susceptible to floods. Moreover, as the previous analysis of policy implementation has shown, the most important factor governing household decisions as to where to locate is the neighborhood quality. Accordingly, we define two policies to increase the neighborhood quality on northern and southern lands adjacent to the current city boundary that are less vulnerable to flood events. Based on Policy I, we build educational facilities as well as shopping centers in northern Boulder. Policy II focuses on building parks and water bodies in southern Boulder. Projections for Policies I and II are illustrated in Fig. 7c–d, revealing that adopting these policies will direct the future development toward the northern and southern regions at Boulder where are less susceptible to future flood scenarios. Table 3 also summarizes the percentage of each development scenario falling within the 500-year floodplain. These results show that if the developer does not consider the risk on the initial analysis, it will result in the highest percentage of future development in floodplains and consequently increases the flood risk in the future.

**Table 3:** Percentage of future residential buildings that are inside the 500-year floodplain.

Scenarios	Percentage of growth inside the floodplains (%)
Normal behavior	16
Risk-informed behavior	7
Policy I	5
Policy II	13

## Discussion

The framework developed in this study enables planners to investigate the role of human behavior in achieving resilient communities susceptible to floods through sustainable development. This framework is a holistic toolset that helps a community to adopt risk mitigation policies that are compatible with human behavior. Such policies will be more acceptable to people than other nonstructural flood mitigation measures, such as acquisition and buyout that require large social and economic investments. The core strength of the proposed framework is the behavioral urban growth model which is the integration of the Relocating Model that simulates the dynamics of urbanization *within* the city boundaries and the Growth Model that mimics the urban expansion *at* the city boundaries. This feature helps us to understand household locational choices.

The analysis of the real estate agent has revealed that, for the testbed used in this study, the housing prices are lower inside the floodplains compared to the prices outside the floodplains, leading to more sale transactions in the floodplains if the buyer agents exhibit Risk-Negligence behavior. Such behavior increases housing choices inside the 100-year and 500-year floodplains to 17% and 29%, respectively, up to 2040. On the other hand, if the buyers exhibit Expected Utility behavior to make risk-informed locational choices, housing choices inside the 100-year and 500-year floodplains will decrease as much as 2% and 15%, respectively. This result emphasizes the need for educating individuals who are seeking housing about flood risk to different properties. Over time, this will reduce the popularity of properties in areas susceptible to floods and allow policymakers and stakeholders to implement measures such as buyout and acquisition with less social and economic costs and political resistance. Neighborhood quality, defined as the accessibility to green spaces, water bodies, highways, education facilities, and the city center favors areas leading to more choices by the Risk-Negligent buyers in such locations. Neighborhood quality is most influenced by accessibility to education centers, highways, the city center, water bodies, and green spaces. These findings encourage planners to adopt socioeconomic incentives, according to this rank, that promote urbanization in less vulnerable areas in floods, leading to reducing future flood risk to the community. One point to note is that this rank order may be different for other cities and the detailed proposed framework in this paper can be used to extract the most influential variables in people decisions on their locational choices and be imposed on the community as socioeconomic incentives to shape urbanization toward building resilient communities in floods. Finally, the developer behavior at the city boundary itself can result in safer communities if flood risk is included in their estimation of their expected return.

All models have limitations, and this model is no exception. For example, in this study, we have not considered the geomorphological and hydrological changes in the region brought about by the combined effect of urbanization and climate change, which could increase the extent of the floodplains^34^. Also, to model the decision under risk we have assumed that the agents’ behavior is rational and make a fully informed decision about their locational choices. Future work will expand the current behavioral study to that of decision-making under limited information. Another avenue of further work could include the role of climate change and how repetitive flooding—learning by experience—may affect the decision of households under risk. Finally, the current study has been performed for the case of flooding as the dominant hazard in the region, but we see no reason the current framework could not be expanded to other hazards as well.

## Conclusions

In this study, we have focused on investigating the effect of human behavior as one of the underlying factors that affects urbanization over time. We also examined how perceptions of flood risk can change the decisions of individuals as to where to locate and what characteristics of a neighborhood entice households to make such decisions. This information was used to weigh the merits of various nonstructural mitigation measures for flooding in terms of socioeconomic incentives to move urbanization toward creating sustainable communities that are resilient to flooding. Our proposed framework employs a behavioral urban growth model that uses ABM to evaluate the effect of human behavior as influenced by urbanization on community vulnerability to floods. The observations from our analysis addressed some of the long-stated challenges in considering the human behavior factors that add non-stationarity in future flood risk and render employed disaster risk reduction measures less effective. The results demonstrated when individuals make an informed decision about their housing choices, housing choices inside the 100-year and 500-year floodplains are reduced by 2% and 15%, respectively. Moreover, under the proper socioeconomic incentives, derived from the preferences of individuals on their housing choices, the percentage of future urban growth of the community inside the 100-year and 500-year floodplains can be reduced from 16% to 5% by the year 2040 for the selected testbed in this study. We aim that this study will initiate a dialogue in a new direction of quantifying the role of human behavior in flood risk assessment of communities. Our framework enables policymakers and local authorities to achieve a more accurate picture of the future of their communities, to employ mitigation measures for protecting cities from flood hazard, and to perform tradeoffs between costs and benefits of future land development in investing in sustainable and resilient communities.

## Material and methods

### Behavioral urban growth model

Urbanization is the process of land-use change that is affected by the interaction of social, biophysical, economic, and political entities^35^. Individuals’ behavior—sellers, buyers, real estate, and developers—and their interactions affect urbanization over time. These behaviors can be influenced by perceptions of flood risk. Modeling human behavior and its effect on the built environment is a complex endeavor as individuals do not make decisions randomly, but decide based on their knowledge, characteristics, and resources^36^. The question that may arise here is that how we can model human behavior. ABM is a technique that can be used to simulate behaviors of autonomous entities and has applications in geographic and urban systems such as pedestrian modeling, traffic simulation, residential dynamics, and urban growth models of cities and regions. This modeling technique provides an opportunity to study the behavior of entities and their heterogeneity on urban systems and their role in shaping their environment^36^.

The employed behavioral urban growth model in this study uses ABM to capture human behavior and its impact influenced by flood risk on urbanization over time. This model consists of two parts: *Relocating Model* which is responsible for simulating the dynamic of urbanization *inside* the current city limit and the *Growth Model* that mimics urban expansion *at* the city boundary over time. These two models are connected through the supply and demand of the real estate market. Some buyers may prefer to live within the city boundary while others select new housing in suburban areas. Thus, the presence of these two sub-models is essential in simulating the urbanization process over time accurately.

#### Relocating model

This model simulates the interaction of real estate, seller, and buyer agents to assess how the interactions of these agents shape the dynamic of urbanization *inside* the city boundary. To do so, the real estate agent provides an estimation of housing prices using a hedonic price model^29^ which is a function of the structural, neighborhood, and environmental characteristics, as below:

1 $${H}_{Trans}=H (s, n, e)$$

The function, ${H}_{Trans}$, is assumed to be a linear function of different variables that range from housing characteristics to proximity to amenities, to hazard level (see Table S1). The coefficients, *β,* are determined by ordinary regression analysis:

2 $$Ln {H}_{Trans}={\beta }_{0}+\sum_{i=1}^{n}{\beta }_{i}{x}_{i}$$

This function is trained using historical datasets from 2010 to 2020. The structural variables used in this model are building age, bedroom number, bathroom number, and square footage. The neighborhood and environmental variables considered are accessibility to highways, green spaces, water bodies, city center, educational facilities, the sale transaction year, and the existence within 100-year or 500-year floodplains. As the simulation proceeds, the successful transaction will be added to re-train the regression and re-assess the coefficients to account for the dynamic of the land market and changes in the housing prices through the years. The last two variables in Table S1, FLD 100 and FLD 500, have been added to the hedonic price model to evaluate how the floodplain presence affects the real estate market. After training the model with the historical dataset, the real estate agent predicts the housing prices in every time step of the analysis. This agent estimates housing prices with and without considering flood risk due to the housing location if it is within or outside the floodplains. More details of the real estate agent can be found in Supplementary Information Section 1.1.1.

The seller agent is the other effective entity in the Relocating Model that participates in this process by providing the housing options and adding them to the real estate market. The seller decides to relocate or leave the study region. Regardless of the incentives, s/he aims to maximize profit. For modeling the seller's behavior, we need to calculate the number of sellers and the location of sale within each time step. The number of sellers is calculated using the yearly number of historical sale transactions within the study region from 2010 to 2020. These numbers are used to determine the mean and standard deviation of a normal distribution. Then, using the distribution characteristics, we generate random numbers as the available housings within the market at any time step. The location of sale is also calculated randomly from the available housing in the study region. More details regarding the seller agent can be found in Supplementary Information Section 1.1.2.

The buyer agent is one of the most critical components of the Relocating Model. Accordingly, a buyer is a household that seeks housing that can maximize its utility. Whether the buyer considers the flood risk on their decision or not, s/he decides where to locate according to two behaviors: Risk-Negligence and Expected Utility. These two behaviors are considered to account for a full range of possible actions from the buyer within the market. Risk-Negligence happens when a household does not consider risk when s/he searches for a property to buy. This point does not mean that s/he is unaware of the risk. In fact, flood risk is not considered as a substantial factor when s/he wants to offer a bid price. Therefore, their decision about the locational choices is limited to housing prices. S/he forms a utility for a property based on hedonic analysis of sale price that is a bundle of structural and neighborhood characteristics^30^. In this scenario, the buyer utility function is calculated as below:

3 $${U}_{0L}={A}_{i}*{X}_{i,norm}$$

where ${X}_{i}$ is a vector consisting of housing characteristics that play a role in locational choices by buyers. In this study, this set includes bedroom number, square footage, building age, and neighborhood quality. Neighborhood quality is the residual of the hedonic price model for each housing and is defined as accessibility to highways, education facilities, city center, water bodies and lakes, and parks and green spaces. Also, ${A}_{i}$ is the coefficient of such characteristics that shows heterogeneity in the preferences of different people on housing features. The summation of ${A}_{i}$ should be 100 indicating that variation in the sale price is considered. In this way, we can consider the heterogeneity in agent behaviors that can make the modeling approach more realistic. The other behavior representative of household decisions under risk is Expected Utility (EU). Initially, this is based on the assumption that the economic actors—households—make a fully informed decision based on the perfect information they have for all of the available housing options in the region. In other words, Expected Utility is based on the assumption that households are fully rational agents. Then, based on this theory, it is assumed that the households form a utility expectation for each housing unit and they select the unit with the highest utility to reach their ideal preferences. To consider the flood risk and decision under a risky situation, the utility for a property in flood-prone areas is calculated based on Eq. (4), as below:

4 $${U}_{L}=-0.25*{U}_{0L}$$

In this equation, the coefficient of 0.25 accounts for the average insurance damage claims which is equal to 25% of the property values^37^. This value serves as a benchmark for the average property loss in the case of flooding which households decide on their choice in buying the property. Moreover, ${U}_{0L}$ represents the utility of a property without considering the risk. To consider the probability of the flooding (${P}_{N}$) in the average length of residence (Yr), which is equal to 10 years in this study, Eq. (5) are used, as below:

5 $${P}_{N}={P}^{N}*{\left(1-P\right)}^{Yr-N}*\left(\genfrac{}{}{0pt}{}{Yr}{N}\right)$$

where, P is the annual flood probability, and N is the number of flooding that can occur within the residence length. In this study, we assume that each property can experience only three flooding events at most, in their life. Then the utility for properties under flood risk is calculated based on Eq. (6), as follows:

6 $$EU=\sum_{N=1}^{3}{U}_{Nloss}*{P}_{N}$$

where,

7 $${U}_{Nloss}=(1-0.25)*{U}_{0L}$$

In Eq. (7), ${U}_{Nloss}$ is a utility gain for a property for a specific number of flood events. Some details of the buyer agent have been represented in Supplementary Information Section 1.1.3.

The real estate, seller, and buyer agents in the Relocating Model interact through the negotiation process. Buyers and sellers set an expectation of their bid and ask prices. To register a successful sale transaction, these expectations should be within a specific threshold. Using the actual housing prices in the market, the real estate agent will provide feedbacks for both buyers and sellers and help to achieve a successful sale transaction. If the buyers and sellers stay in the market and experience unsuccessful sale transactions, they adjust the expectations of their bid and ask prices to maximize their utility and their profit, respectively. Comprehensive details of the negotiation process are explained in Supplementary Information Section 1.1.4.

#### Growth model

This model mimics the dynamic of urbanization *at* the city boundary resulting from converting the undeveloped to the developed lands and expanding the city boundary. This process occurs because of interaction between the developer, real estate, and buyer agents. The real estate and buyer agents are identical to the relocating agent model. On the other hand, the developer agent is responsible for providing the housing options as a result of buying undeveloped lands and covert them to developed areas to maximize its profit. For modeling the developer agent behavior, we use a Cellular Automata, described in detail in Supplementary Information Section 1.2.1. Cellular Automata (CA) is a powerful geo-simulation tool that has been previously used to model complex geographical systems with nonlinear and evolving characteristics. It uses a set of maps—suitability, accessibility, zoning, and neighborhood maps—in a 2-D spatial rectangular grid to calculate the transition potential as the probability that defines the evolution in time. A transition probability vector is determined within each time step for each cell using the probabilistic function, presented in Eq. (8)^38^.

8 $${P}_{k}^{t}=\vartheta \times \left({A}_{k}^{t}\right)\times \left({S}_{k}^{t}\right)\times \left({Z}_{k}^{t}\right)\times \left({N}_{k}^{t}\right)$$

in which, ${{\varvec{A}}}_{{\varvec{k}}}^{{\varvec{t}}}$ is accessibility to the transportation network, ${{\varvec{S}}}_{{\varvec{k}}}^{{\varvec{t}}}$ is intrinsic suitability, ${{\varvec{Z}}}_{{\varvec{k}}}^{{\varvec{t}}}$ is the zoning status, and ${{\varvec{N}}}_{{\varvec{k}}}^{{\varvec{t}}}$ is the neighborhood effect of the interested cell for land-use k at time t. The parameter $\boldsymbol{\vartheta }$ is the scalable random perturbation number at time t. Calculations for these terms can be found in Supplementary Information Section 1.2.1.

Here, we modified Eq. (8) to account for the role of developer agent, as shown in Eq. (9). In this equation, the developer preferences are considered as a map adding to the set of suitability, Z# accessibility, zoning, and neighborhood maps to examine the role of the developer agent and its preferences both in a normal situation and by considering flood hazard if the developer agent considers flood risk in its expected return.

9 $${P}_{k}^{t}=\vartheta \times \left({A}_{k}^{t}\right)\times \left({S}_{k}^{t}\right)\times \left({Z}_{k}^{t}\right)\times \left({N}_{k}^{t}\right)\times \left({D}_{k}^{t}\right)$$

where ${{\varvec{D}}}_{{\varvec{k}}}^{{\varvec{t}}}$ is the developer map for land-use k at time t. The process of assessing the developer map with and without considering flood risk and the model implementation is thoroughly explained within Supplementary Information Sections 1.2.2 and 1.3, respectively.

### Flood hazard module

The flood hazard in this study is measured by its frequency, intensity (water depth and velocity), and the extent of flooding, known as the floodplain. We use the coupled hydrologic-hydraulic analysis in the flood module of HAZUS-MH^39^, a loss estimation platform developed by the Federal Emergency Management Agency to calculate the floodplain characteristics for various flooding scenarios, including 100- and 500-year return periods. Based on a review by Banks et al.^40^, HAZUS-MH was identified as the best tool for the flood damage assessment compared to other available tools such as MIKE flood, water RIDE, hydrologic engineering center flood impact analysis (HEC-FIA). In addition, Tate et al.^41^ suggested that the results obtained from the default hydraulic analysis in HAZUS-MH are suitable for regional analysis, which further confirmed the suitability of using HAZSU-MH in our study since we focused on regional analysis as well. It should be noted that uncertainties in the floodplain characteristics calculated by HAZUS-MH are large due to the simplifications in the model. Moreover, changes in surficial geology and hydrology due to urbanization are not considered^11^. Some studies (e.g., Gori et al.^34^) have quantified these changes by coupling more detailed hydrologic-hydraulic software, such as HEC-HMS^42^ and HEC-RAS^43^ to land-use projection models. However, since the objective of this study is to investigate the effect of human behavior on urbanization and exposure to risk and to introduce a methodology for evaluating different nonstructural strategies in terms of socioeconomic incentives and land-use policies, HAZUS-MH is sufficient for our purposes.

### Policy implementation module

In this study, we focus on nonstructural flood mitigation measures in terms of socioeconomic incentives that are designed to be compatible with human behavior. The behavioral urban growth model reveals information about the critical factors in people’s decisions on their locational choices. This information is used to design socioeconomic strategies encouraging households to the safer location gradually with time. As the developer agent is responsible for simulating the growth of the community over time, the first two scenarios, Normal Behavior and Risk-informed Behavior, are considered to investigate the behavior of the developer agent behavior on urbanization. Additionally, we define the Policy I and Policy II scenarios according to the information revealed in the statistical analysis to be applied, as the future development plans, by the developer agent to shape the communities moving toward resilience. Each of the development scenarios is explained below:

- *Normal behavior: *This policy is designed to evaluate the developer's behavior when the agent does not consider the flood risk on their decision. Accordingly, the developer preferences are considered using a map calculated by Equations S6 and S7. This scenario simulates the situation where the developer agent does not consider the flood risk on how the city expands through years and how the developed areas are selected by buyers.
- *Risk-informed behavior:* This policy is defined to evaluate how the consideration of flood risk by developer agent will change the city expansion over time. Equations S6 and S9 are used to assess the developer map and expected return based on this scenario.
- *Policy I: *As the statistical analysis has revealed, accessibility to educational facilities is the most influential aspect of buyers’ decision on where to locate. After that, commercial facilities, highways, parks and water bodies are important the most. Based on Policy I, we build educational facilities as well as shopping centers in northern Boulder to promote urbanization in this direction. These incentives will affect the suitability term of Eq. (9).
- *Policy II: *Policy II focuses on encouraging urbanization in the southern part of Boulder by constructing parks and lakes. This will also impact this process by increasing the suitability of southern Boulder where is less susceptible to flood events compared to eastern Boulder. Again, these incentives will affect the suitability term of Eq. (9).

## Supplementary Information

Supplementary Information.

## Data Availability

The data that supports the findings of this study are available from the corresponding author upon request. The Digital Elevation Model (DEM) input for HAZUS-MH software can be obtained from (https://viewer.nationalmap.gov/basic/). The land-use maps and geospatial information were obtained from the City of Boulder and OpenStreetMap (http://www.openstreetmap.org) that are already provided in this study.

## Code availability

The model components have been described in the paper and Supplementary Information. The code required for analysis are available from the first author and corresponding author on request.
