# Supplementary material for: Unraveling the complexity of human behavior and urbanization on community vulnerability to floods
Source: Sci Rep. 2021 Oct 11;11:20085. doi: 10.1038/s41598-021-99587-0 (PMC8505605; doi:10.1038/s41598-021-99587-0)
Supplement: Supplementary file 1 — Supplementary Information. [file 41598_2021_99587_MOESM1_ESM.pdf]

Unraveling the complexity of human behavior and urbanization on community vulnerability to floods

Mona Hemmati[1,2], Hussam N. Mahmoud[2,*], Bruce R. Ellingwood[2], Andrew T. Crooks[3]

[1]Lamont-Doherty Earth Observatory, Columbia University, Palisades, NY, USA
[2]Department of Civil and Environmental Engineering, Colorado State University, Fort Collins, CO, USA
[3]Department of Geography, University at Buffalo, Buffalo, NY, USA
*Corresponding author: Hussam N. Mahmoud (Hussam.Mahmoud@colostate.edu)

SUPPLEMENTARY INFORMATION

This PDF file includes:

Behavioral Urban Growth Model
Effect of Flood Risk on Agents' Behaviors
Figures S1 to S8
Table S1
References

1. Behavioral Urban Growth Model

1.1 ***Relocating Model.*** The Relocating Model, as the first sub-model of the behavioral urban growth model, reproduces the urbanization dynamic within the city boundary. The three agents available in this sub-model are real estate, seller, and buyer agents. The flowchart for the Relocating Model is represented in Figure S1. In this section, we provide more details for these agents.

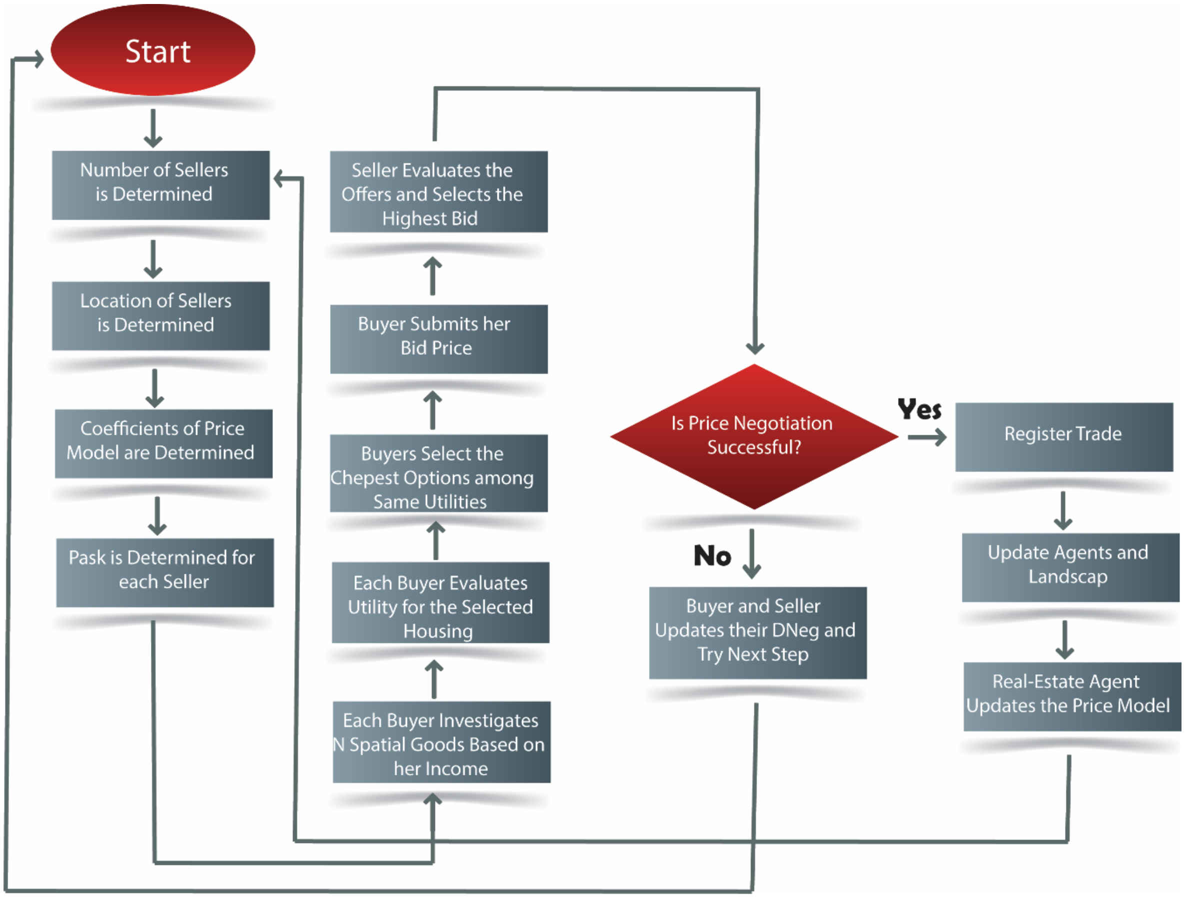

Figure S1. Framework for the Relocating Model of the behavioral urban growth model proposed in this study.

1.1.1 Real estate Agent. Real estate agents are responsible for providing estimates of housing prices at every time step of the analysis and usually participate in the negotiation process to facilitate the transaction between buyers and sellers. They predict the housing price using a hedonic price model (Bin et al., 2008), in which "The basic concept is that differential property prices reflect the way a household values different real property characteristics. Residential properties have a variety of attributes and observing how property values change as these attributes, including square footage, acreage, and flood hazard exposure, change provides a way of estimating the incremental value of these attributes to property owners" (Bin et al., 2008). The variables considered in the hedonic price model are presented in Table S1.

Table S1. Considered variables in hedonic price model used by real estate agent

Variables	Description
HWY	Distance to the closest highway
PARK	Distance to park and green space
WATER	Distance to waterbody and river
CBD	Distance to city center and downtown
EDU	Distance to educational centers
AGE	Building Age
BED_RM	Bedroom Number
BATH_RM	Bathroom Number
SQFT	Square Footage
YEAR 2010-2015	Year of sale transaction (Dummy variable)
FLD 100	Existence of 100-year floodplain (Dummy variable)
FLD 500	Existence of 500-year floodplain (Dummy variable)

1.1.2 Seller Agent. Seller agents form price expectations within the real estate market. They affect the housing market by setting ask prices for the housing options that are inside the city boundary. Sellers may decide to put their property in the market for sale for a number of reasons – changes in employment, a need for more space, or greater utility. They can relocate within the region or move to another urban area. Regardless of their motivation, they seek to maximize their profit. The sellers' behavior is modeled using the methodology proposed by Filatova (2015). Two parameters are calculated initially to model the seller's behavior: the number of sellers and the locations of their property. The total numbers of seller agent available in the model is assessed by generating a random number using a normal distribution, in which the mean equals the fraction of properties on the market in each year and the standard deviation that is defined exogenously based on historical data of sale transactions.

At the initialization stage, the seller sets an ask price (P_{ask}) using the same hedonic price model as that used by the real estate agent. However, as the model evolves in time and new sales are recorded, the price is revised considering these new transactions. Sellers also participate in a transaction negotiation process when they consider the feedback from the real estate agent, the duration that their property is on the market, and the number of unsuccessful attempts to sell it. In this proposed behavioral urban growth model, we assume that frequency and intensity of floods may be one of the reasons motivating the sellers to relocate, although evaluating how much this may affect the household's decision to move will not be considered.

1.1.3 Buyer Agent. Buyers represent the households who are seeking a property to maximize their utility. Buyers select a property based on their preferences and budget. They can choose from either newly developed homes at or outside the city boundaries or homes within the city boundaries which are in the market by sellers. Buyers are heterogeneous, in the sense that their behavioral characteristics, amenities, preferences, income, and budget are different. This disparity results in a diversity of decisions. Buyers form their expectations of the home price dynamically based on their preferences over time. Accordingly, they may not consider flood risk as a factor in their decision or they may be unaware of it when they decide on their housing choices. Based on a study by Chivers and Flores (2002), in some areas like Colorado where the region is susceptible to riverine or flash flooding, the majority of people do not consider flood risk as a critical factor. In contrast, people living in hurricane-prone coastal areas like the Southeast or Gulf Coast of the U.S. are more aware of flood hazards, insurance, and the concept of floodplains (Bin et al., 2008). The locational choices can range from objective judgment by perfectly rational agents to subjective judgment under bounded rational behavior to a more cognitively complex psychological model (De Koning, 2017). To consider this wide range of buyers' behavior in this study, we used the two types of behaviors (De Koning, 2017): Risk-Negligence and Expected Utility. These behaviors are explained thoroughly in the main manuscript in the Material and methods section.

1.1.4 Negotiation Process. To complete a successful transaction in the housing market, the buyer's bid price must be within an acceptable range of the seller's ask price. After a buyer finds a home that maximizes his/her utility, s/he

submits an initial bid price which may change over time as a result of negotiation with the seller. The bid price can change over time depending on many factors, such as the duration that a property is in the market, the number of buyers available in the market, and relative market power. A previous study (Filatova, 2015) has suggested that the buyer's bid price is usually fixed between 3% to 5% below the ask price but maybe up to 7% to 10% of the ask price for either an aggressive transaction or if the property has been on the market for a long period.

At the same time, the seller's ask price depends on past unsuccessful negotiations, the time that a property is in the market for sale, and the number of buyers. Sellers typically remember the number of past unsuccessful negotiations, N_{USTr}, and set a threshold, $D_{Neg-seller}$, defined as the difference between the ask price and the highest submitted bid, during the price negotiation procedure. For example, if the seller can afford one month of mortgage payments and does not receive a satisfactory bid price, he may choose to stay in the market for another month. Therefore, $D_{Neg-seller}$ at the beginning of the trading period equals one month of his mortgage, based on his home price, when he bought it, H_{trans}. As the simulation proceeds and the number of unsuccessful attempts increases, D_{neg} is calculated as below:

$$D_{Neg-seller} = kH * H_{trans}/12 * (1 + N_{USTr}) \quad \text{(S1)}$$

where kH converts the property price determined by the real estate agent to an annual payment and $D_{neg-buyer}$ equals one-month of the average rent in the region. The idea is that if the buyer can afford the one month rent, he stays in the market and searches for a better property.

The negotiation process takes place among buyer and real estate agent to submit a bid price, seller and real estate agent to submit an ask price, and buyer and seller to shape the final sale transaction. The bid and ask prices can change over time based on the feedback that buyers and sellers receive from the market. These behaviors all shape the land market, and consequently urbanization; therefore, they should be part of the modeling process (Filatova, 2015), as shown in Figure S2.

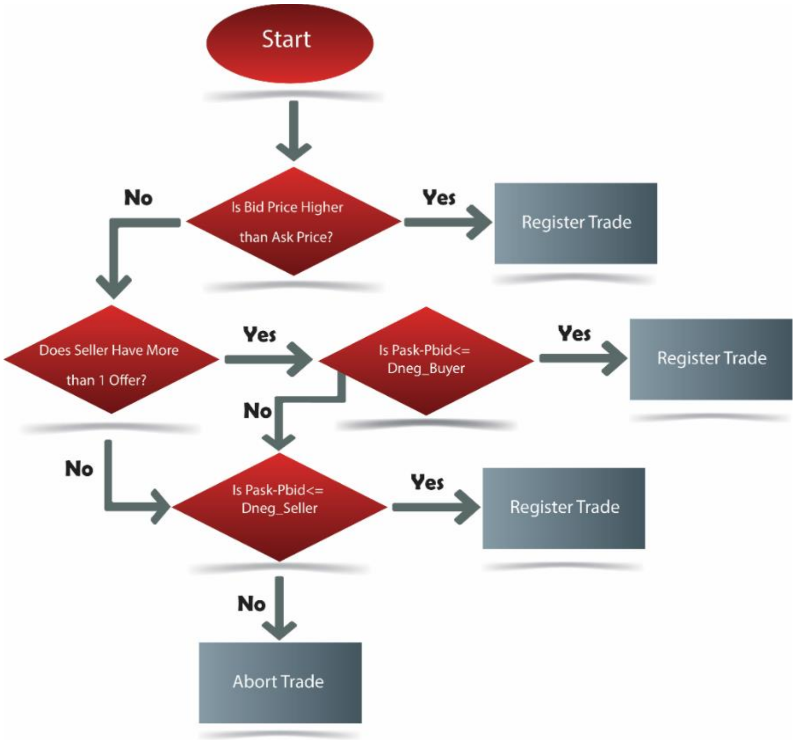

Figure S2. Framework for the negotiation process for behavioral urban growth model, following Filatova, 2015.

1.2 Growth Model. The second sub-model used in this framework is the Growth Model. This model is responsible for simulating urban expansion at the city boundary as a result of converting undeveloped to developed land by developer agent. The characteristics of the Growth Model and how it has been modified to capture the developer agent behavior are explained subsequently.

1.2.1. Cellular Automata (CA). The growth model employs a Cellular Automata (CA) approach (White et al., 1997; Barredo et al., 2004) that is characterized by the following elements, as illustrated in Figure S3:

(a) ***Cell Space:*** is a 2-D spatial rectangular grid over the study area consist of different cells with a considered cell size of $100\ m \times 100\ m$ for representing a residential neighborhood.

(b) ***Cell State:*** each cell has its state serving as the land-use pattern of the cell (e.g. developed or undeveloped). The state of a cell can be either passive or active. The former does not contribute to the urbanization process while it affects this process by adding favorable or unfavorable incentives to surrounding areas. The latter does contribute to the urban expansion process and the state of such cells can change through time. For the purpose of analysis in this manuscript, the fixed land-use classes are considered as roads, airports, water bodies, and abandoned lands. Moreover, considered active cell states are residential, commercial, industrial, and vacant lands.

(c) ***Neighborhood:*** is the impact that the adjacent or surrounding cells have on a cell in the growth process. The neighborhood characteristics are quantified using a specific shape and size. Herein, as shown in Figure S3, the neighborhood is a circular region with its center located at the cell of interest and a radius of 8 surrounding cells.

(d) ***Time:*** is a discrete parameter in this proposed CA growth model. In this study, we considered annual increments to reflect the city expansion on yearly basis.

(e) ***Transition Potential:*** is the probability that a cell reaches a specific state at time step t. This probability is a function of a *perturbation factor, accessibility, suitability, zoning status, neighborhood* effects as represented in Equation S2.

$$\boldsymbol{P_k^t} = \boldsymbol{\vartheta} \times (\boldsymbol{A_k^t}) \times (\boldsymbol{S_k^t}) \times (\boldsymbol{Z_k^t}) \times (\boldsymbol{N_k^t}) \tag{S2}$$

In Equation S2, $\boldsymbol{A_k^t}$ is accessibility to the transportation network, $\boldsymbol{S_k^t}$ is intrinsic suitability of a cell representing how favorable the cell is for future development, $\boldsymbol{Z_k^t}$ is the zoning regulation adopted by the City, and $\boldsymbol{N_k^t}$ is the neighborhood effect of the surrounding environment on the growth of an interested cell for land-use k at time t. Here, the perturbation factor $\boldsymbol{\vartheta}$ is the scalable random number that accounts for uncertainty in the urbanization process

$$\boldsymbol{\vartheta} = \mathbf{1} + (-\boldsymbol{ln}\ \boldsymbol{R})^{\boldsymbol{\alpha}} \tag{S3}$$

where **R** is a random variable uniformly distributed between [0,1] and $\boldsymbol{\alpha}$ is a parameter that calibrates the perturbation number.

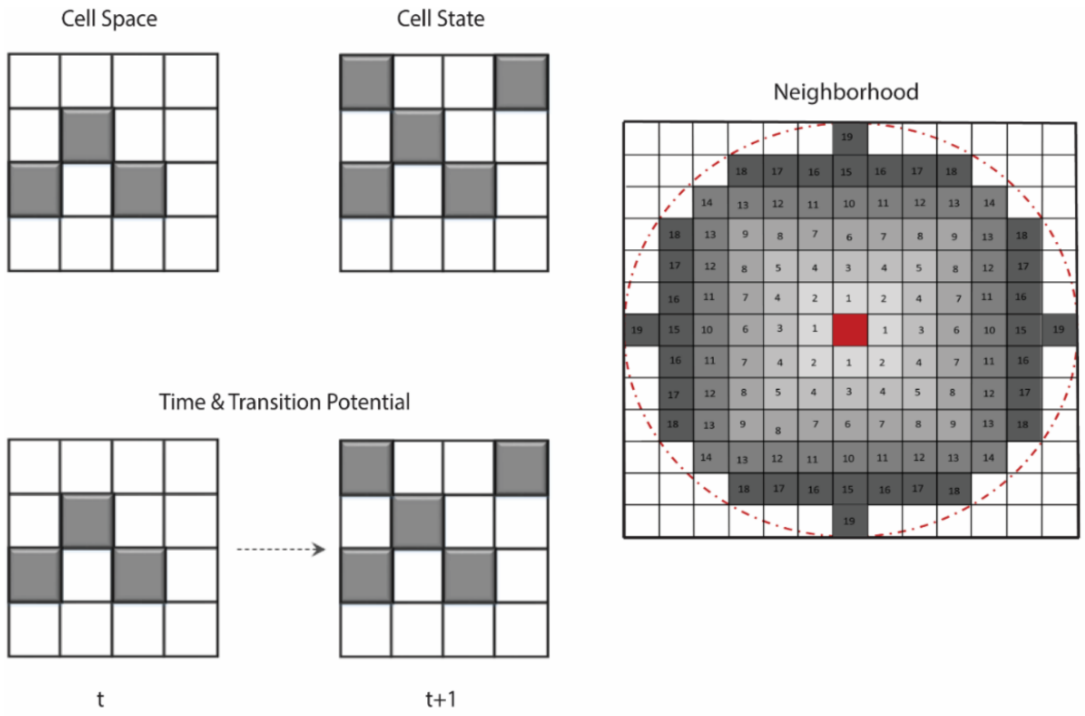

Figure S3. Components of the Growth Model used in the behavioral urban growth model in this study.

The accessibility, suitability, zoning, and neighborhood terms should be assessed for each cell in every time step. Accessibility evaluates how significant access to the transportation systems is for the growth of the cell for various types of occupancies. On that note, commercial and industrial occupancies rely more on accessibility to roads. Therefore the accessibility term of the transition probability has a higher impact on future developments. The accessibility term for each cell and time step is quantified by Equation S4:

$$\boldsymbol{A_k^t} = \left(\mathbf{1} + \frac{\boldsymbol{D_r}}{\boldsymbol{a_{r,k}}}\right)^{-1} \tag{S4}$$

where $\boldsymbol{D_r}$ is the Euclidian distance of a cell to the nearest road and $\boldsymbol{a_{r,k}}$ is the calibrated distance-decay accessibility coefficient which reflects the importance of road access for land-use *k* (Barredo et al. 2004).

The second term of the transition potential is the *suitability* of the cell that represents the favorability of a cell to a specific land-use type. Herein, we consider some features, including land price, green land, water bodies, and other opportunities for recreation, educational centers, schools and universities, and general public facilities as the factors that contribute to the urbanization process. This term is assessed by calculating the Euclidian distance of a cell to the aforementioned physical features using their geographic coordinates.

The third term in transition potential is the *Zoning* term that uses the development plan and zoning maps to set a binary value of zero and one to each cell indicating whether a certain land-use is permitted for development.

The last term in Equation 2 is the *neighborhood*. The concept is that the cell with a larger distance to the interested cell has a lower weight on the growth of the cell and vice versa. These weights are calculated by the *neighborhood* term for each cell and land-use using Equation S5. For more information regarding this model, you can refer to (Hemmati et al., 2021).

$$\boldsymbol{N_k^t} = \sum_{c}\sum_{l} \boldsymbol{w_{k,L,c}}\,{}^{t}\boldsymbol{I_{c,L}} \tag{S5}$$

where $\boldsymbol{w_{k,L,c}}^{\boldsymbol{t}}$ is a weighting parameter expressing the strength of the interaction between a cell with land-use L at a distance c from the cell of interest in the neighborhood; $\boldsymbol{I_{c,L}}$ is an indicator function in which $\boldsymbol{I_{c,L}} = \boldsymbol{1}$ if cell l at a distance c is in state L and $\boldsymbol{I_{c,L}} = \boldsymbol{0}$ otherwise (Barredo et al., 2004).

1.2.2. Developer Agent. The developer agent is responsible for modeling regional growth at the urban boundaries. This agent acts as the mediator between farmers and/or government agencies owning undeveloped land at the city boundary and household agents seeking a property to develop that will maximize their utility (Parker and Filatova, 2008). Developer agents purchase undeveloped lands from landowners, arrange for utility services, construct housing, and supply these newly developed entities to the real estate market. The developer also observes the competitive bidding process among consumers for the existing building stock, and forms expectations of future prices based on observed prices. The developer then extrapolates price expectations to all undeveloped locations on the landscape. Developer demand for land is calculated as the difference between expected future population growth and the combination of currently vacant houses and owned inside the city boundary. If demand exceeds currently available housing capacity, ask prices for each undeveloped land parcel are formed based on these price expectations, net of construction costs, and carry cost (Magliocca, 2017).

The role of the developer agent is modeled using Equation S6, which is similar to Equation S2 but includes additional term for developer agent behavior. The developer preferences are considered as a map, adding to the set of suitability, accessibility, zoning, and neighborhood maps, to represent the role of developer agent and its preferences both in a normal situation and by considering flood hazard.

$$\boldsymbol{P_k^t} = \boldsymbol{\vartheta} \times (\boldsymbol{A_k^t}) \times (\boldsymbol{S_k^t}) \times (\boldsymbol{Z_k^t}) \times (\boldsymbol{N_k^t}) \times (\boldsymbol{D_k^t}) \qquad \text{(S6)}$$

where $\boldsymbol{D_k^t}$ is the developer agent behavior for land-use k at time t, explained in the following section.

The developer uses the expectation of land price and demand information to calculate his/her profit expectation. The developer map ($\boldsymbol{D_k^t}$) is calculated for normal conditions using Equation S7:

$$\boldsymbol{D_k^t} = (1 - r) * \frac{(E\langle L_{Price}|k, t\rangle - C_{cost})}{Z} - \frac{C_{carry_{t-1}}}{A_{d,t}} \qquad \text{(S7)}$$

where $\boldsymbol{D_k^t}$ is the expected return from each housing type k and time t, $E\langle L_{Price}|h, i, t\rangle$ the expected land is return, calculated using Equation S8 (described below), r is the discount rate (assumed to be 5%), C_{cost} net construction and infrastructure expenses for each housing type, $C_{carry_{t-1}}$ is the cost to the developer of holding a vacant property for one year, Z is a coefficient that converts the expected return per lot to expected return per acre, and $A_{d,t}$ is acre demand at time t.

The developer agent must have an expectation of land prices in the future to estimate his/her profit. In this study, we use a hedonic price model explicitly trained for the developer agent to predict the land price in the future. To do so, we consider some key factors playing a role in land price, including median household income, lot size for any given housing type, travel cost, distance to educational centers, water bodies, green spaces, parks, and roads. Based on these key factors and using Equation S8 along with historical data from the constructed housing within the city boundary, the price estimation model for undeveloped land is:

$$E\langle L_{Price}|k, t\rangle = \beta_0 + \sum_{i=1}^{n} \beta_i x_i \qquad \text{(S8)}$$

To consider the flood risk on the developer preferences for converting undeveloped lands to developed parcels, Equation S7 is modified to:

$$\boldsymbol{D_k^t} = (1-r) * \frac{(E\langle L_{Price}|k,t\rangle - C_{cost})}{Z} - \frac{C_{carry}{}_{t-1}}{A_{d,t}} - E\langle Loss|k,t\rangle \quad \text{(S9)}$$

where, $E\langle Loss|k,t\rangle$ is the expected flood loss for each housing type k at time t for each cell. Therefore, using Equations S9 and S11, two separate maps are calculated - one that does not consider flood risk and that does - and are used in the CA simulation model to mimic urban expansion by accounting for the developers' preferences.

1.3. Model Implementation. To assess how much of the undeveloped lands will be converted to the developed lands in the future, we need to estimate the future demand. In this study, demand is defined as the number of undeveloped cells that are transformed into developed cells throughout a year. We estimate the demand using the population prediction of the City of Boulder for the year 2040 divided by the average population of each cell. The year 2040 was selected because (1) the CA model provides a better prediction for the near term future since it assumes the past is representative for the future; (2) we have a prediction for population statistics for the region in 2040 which is 123,000 people (Boulder floodplain factsheet, 2018); and (3) urban planning usually is performed for the near-term horizon due to political, economic, and social considerations. Since our objective is to investigate changes in urbanization brought about by nonstructural measures, we found that a 20-year time horizon is reasonable for the urban growth projections after consulting with experts in the field. For more information regarding this model, you can refer to (Hemmati et al., 2021).

To simulate the growth of a community over time, the flowchart presented in Figure S4 should be implemented, as below:

1) For each cell accessibility, suitability, zoning, and neighborhood, developer terms are calculated at a given time step.
2) The transition probability is calculated for each land-use pattern using Equation S6.
3) At each time step, a Monte Carlo technique is used with *n* = 100 to account for uncertainties in the urbanization process. For each simulation, the transition probability is calculated and the growth is estimated.
4) After generating 100 urban growth projection samples, the final projection for the time step is calculated by taking an average from the generated projections in the previous step.
5) The average final projection is the basis for the next time step.

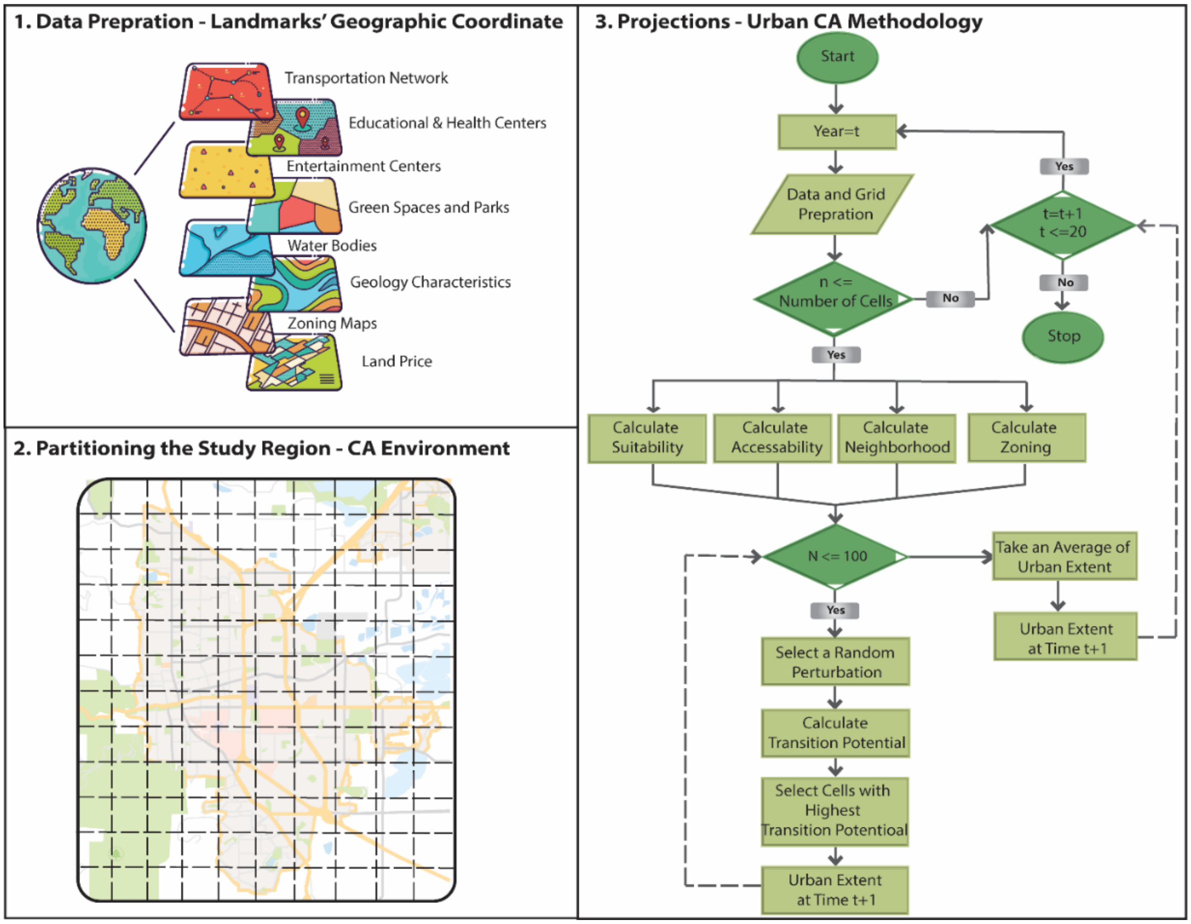

Figure S4. The detailed description of the Growth Module used to simulate the urbanization process over time.

2. Effect of Flood Risk on Agents' Behavior

Figures S5 and S6 show the effects of flood risk on the real estate agent on a historical basis from 2010-2020 and future projections for 2020-2040 for the case study of the City of Boulder. As these figures demonstrate, the housing market inside both 100-year and 500-year floodplains is generally lower compared to the housing prices outside the floodplains.

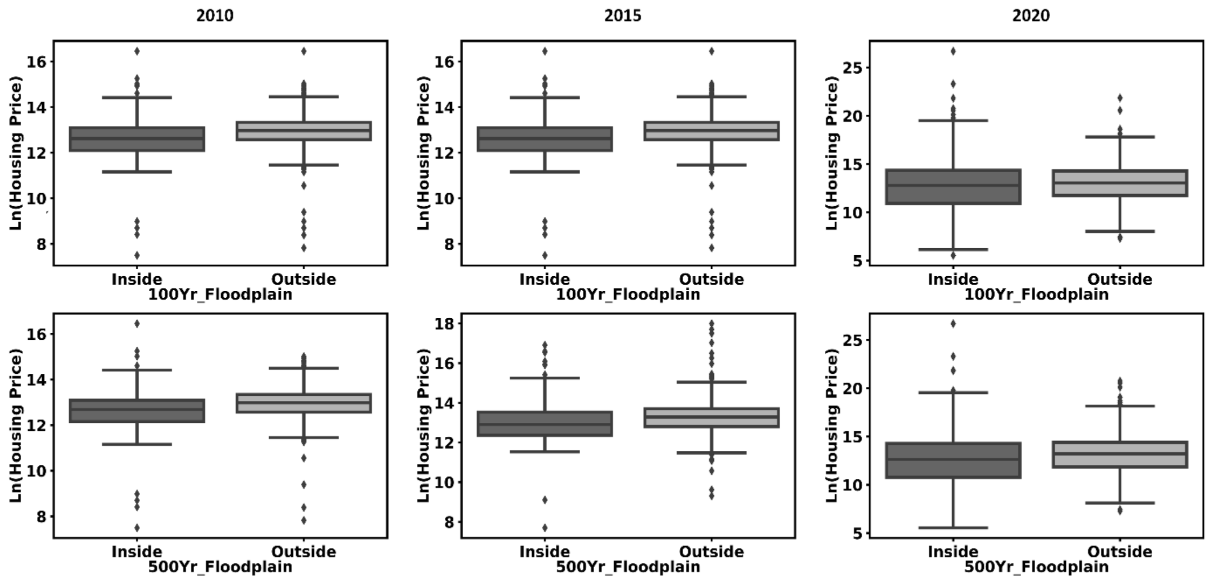

Figure S5. Historical sale transaction from 2010 to 2020 for housing inside and outside 100-year and 500-year floodplains.

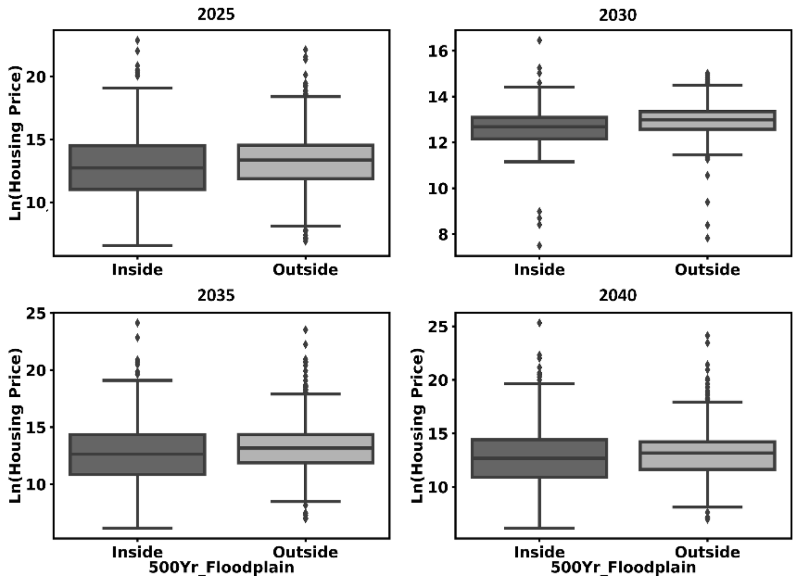

Figure S6. Projection of sale transactions from 2020 to 2040 for housing inside the 500-year floodplain.

Figures S7 illustrate the housing choices on a historical basis from 2010-2017 by buyers exhibiting Risk-Negligence behavior and Expected Utility behavior. Since eastern Boulder is susceptible to flooding and has lower housing prices than northern Boulder, the majority of choices are made by Risk-Negligent buyers. However, the Expected Utility buyers selected less vulnerable regions such as northern Boulder.

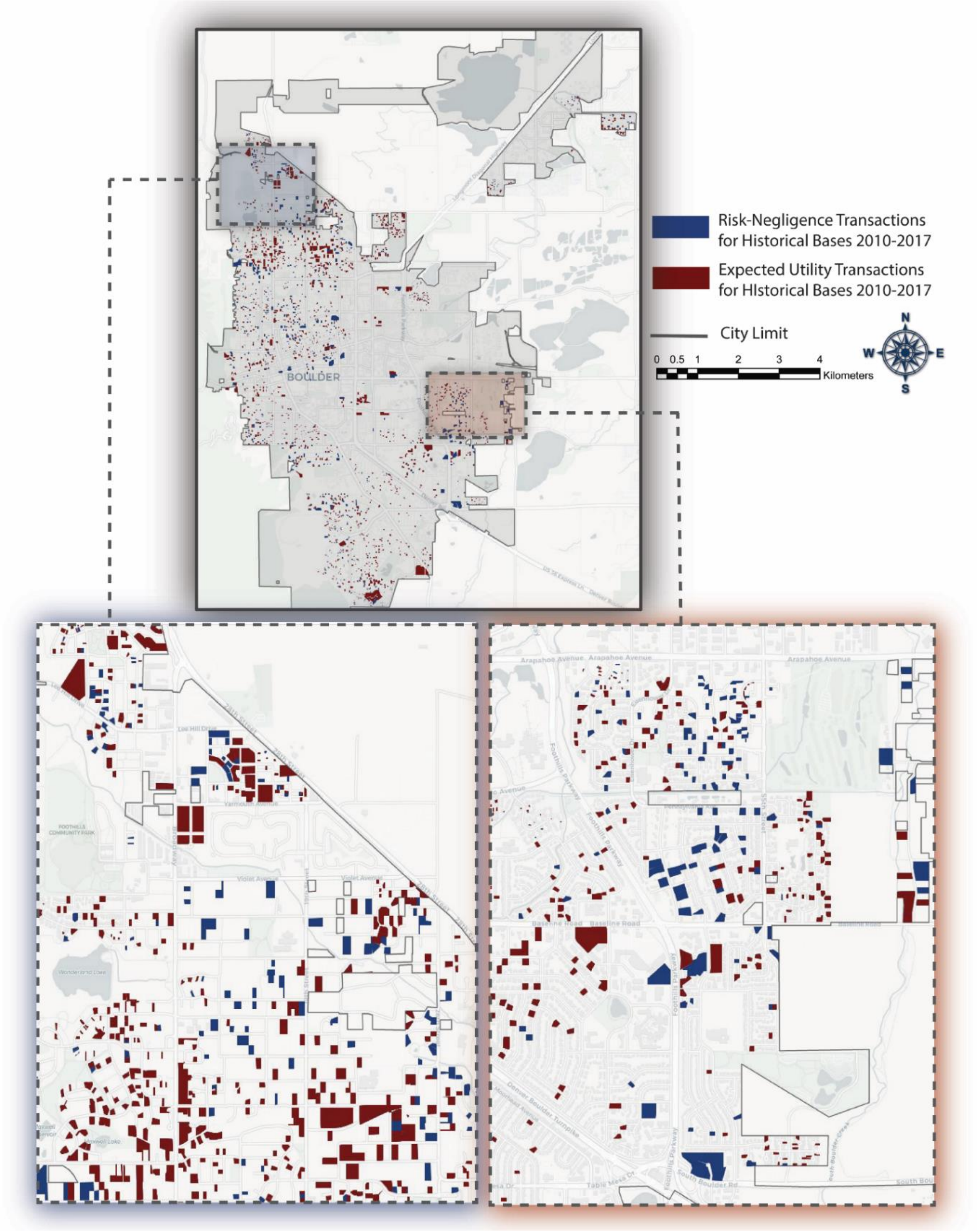

Figure S7. Buyers' choices on a historical basis from 2010 to 2017.

Figure S8 represents the eigenvalue analysis and proportion of variance associated with each principal component. As explained in the main manuscript, to identify the number of principal components, a horizontal line is drawn at an eigenvalue equal to one and the number of components that have an eigenvalue larger than one equals the number of main principal components. Therefore, this figure shows, the housing price problem in this study consist of two main Principal Components (PC1 and PC2) which are responsible for about 40% and 30% of the variation in housing prices,

respectively. The relation between these two main PCs and important characteristics in housing price can reveal the significant factors in individuals' decisions on their locational choices under Risk-Negligence Behavior.

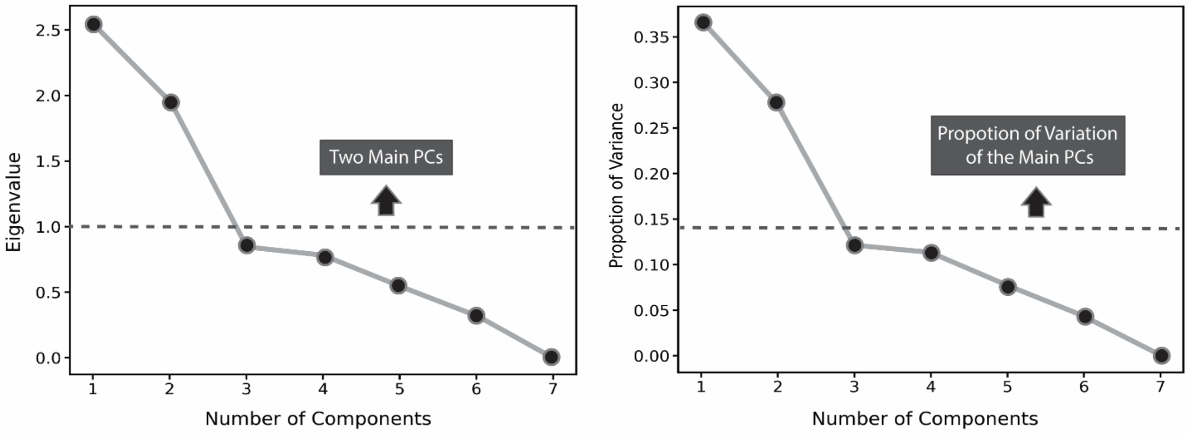

Figure S8. Eigenvalue analysis and proportion of variance for determining the principal components.

3. References

Barredo, J. I., Demicheli, L., Lavalle, C., Kasanko, M., & McCormick, N. (2004). Modelling future urban scenarios in developing countries: an application case study in Lagos, Nigeria. *Environment and Planning B: Planning and Design*, 31(1), 65-84. https://doi.org/10.1068/b29103

Bin, O., Kruse, J. B., & Landry, C. E. (2008). Flood hazards, insurance rates, and amenities: Evidence from the coastal housing market. *Journal of Risk and Insurance*, *75*(1), 63-82. https://doi.org/10.1111/j.1539-6975.2007.00248.x

Boulder Floodplain Factsheet (2018). https://www-static.bouldercolorado.gov/docs/Flood_Fact_Sheet-1201801111508.pdf?_ga=2.237970752.1166715059.1581900748-1258262908.1578502250

Chivers, J., & Flores, N. E. (2002). Market failure in information: the national flood insurance program. *Land Economics*, *78*(4), 515-521. https://www.jstor.org/stable/3146850

De Koning, K., Filatova, T., & Bin, O. (2017). Bridging the gap between revealed and stated preferences in flood-prone housing markets. *Ecological economics*, *136*, 1-13. https://doi.org/10.1016/j.ecolecon.2017.01.022

Filatova, T. (2015). Empirical agent-based land market: Integrating adaptive economic behavior in urban land-use models. *Computers, environment and urban systems*, *54*, 397-413. https://doi.org/10.1016/j.compenvurbsys.2014.06.007

Hemmati, M., Mahmoud, H. N., Ellingwood, B. R., & Crooks, A. T. (2021). Shaping urbanization to achieve sustainable communities resilient to floods. *Environmental Research Letters*. https://doi.org/10.1088/1748-9326/ac1e3c

Magliocca, Nicholas (2017). Coastal Coupled Housing and Land Markets (C-CHALMS) (Version 1.0.0). *CoMSES Computational Model Library*. https://www.comses.net/codebases/5637/releases/1.0.0/

Parker, D. C., & Filatova, T. (2008). A conceptual design for a bilateral agent-based land market with heterogeneous economic agents. *Computers, Environment and Urban Systems*, 32(6), 454-463. https://doi.org/10.1016/j.compenvurbsys.2008.09.012

White, R., Engelen, G., & Uljee, I. (1997). The use of constrained cellular automata for high-resolution modelling of urban land-use dynamics. *Environment and Planning B: Planning and Design*, 24(3), 323-343. https://doi.org/10.1068/b240323
